# 6:2 Cl-PFESA induces sex-dependent lung inflammation through NLRP12 inflammasome activation and pulmonary microbiota dysbiosis

**DOI:** 10.3389/fimmu.2026.1939840

**Published:** 2026-10-30

**Authors:** Suliman Khan, Min Chen, Mubbashar Abbas, Zhen Chen, Zhiwei Liang, Rizwan Ullah, Shuyu Xie, Dongmei Chen

**Affiliations:** 1 National Reference Laboratory of Veterinary Drug Residues (HZAU), Wuhan, China; 2 Department of Veterinary Physiology and Biochemistry, Lasbela University of Agriculture, Water and Marine Science, Uthal, Pakistan; 3 National Key Laboratory of Agricultural Microbiology, College of Veterinary Medicine, Huazhong Agricultural University, Wuhan, China; 4 State Key Laboratory of Agricultural Microbiology Core Facility, Huazhong Agricultural University, Wuhan, China; 5 Hubei Hongshan laboratory, Wuhan, China

**Keywords:** 6:2 Cl-PFESA, lung inflammation, lung microbiota, NLRP12 inflammasome, sex-dependent toxicity, transcriptomics

## Abstract

Widespread detection of 6:2 chlorinated polyfluoroalkyl ether sulfonic acid (6:2 Cl-PFESA), a major substitute for perfluorooctane sulfonate (PFOS), has been reported in various environmental and human biological samples. Yet, its lung inflammation and sex-dependent effects have not been studied. In this study, mice were orally gavaged a single dose of 10 mg/kg/BW 6:2 Cl-PFESA or vehicle control for 28 days. Males exhibited greater susceptibility than females, as evidenced by earlier body weight loss, more severe lung histopathology, elevated inflammatory cytokine expression, and increased pulmonary lipopolysaccharide accumulation. The transcriptomic analysis also showed extensive sexual dimorphism, with a significantly larger number of differentially expressed genes in the female lungs compared with males, with minimal overlap between sexes. The NOD-like receptor signaling pathway was identified as a major responsive pathway, with upregulation of *NLRP12* in both sexes and sex-specific down-regulation of Interferon regulatory factor 7 (*Irf7*) in males and Bcl-2-like protein 1 (*Bcl2l1* (*Bcl-xL*) in females. *In vitro* siRNA-mediated knockdown of *NLRP12* in A549 and THP-1 cells confirmed its role as a positive mediator of the 6:2 Cl-PFESA-induced inflammatory response. Furthermore, 6:2 Cl-PFESA promoted NLRP12 inflammasome-associated activation, characterized by enhanced NLRP12 expression, caspase-1 cleavage, mature IL-1β processing, increased IL-18 secretion, and elevated caspase-1 enzymatic activity. Notably, 6:2 Cl-PFESA significantly altered the lung microbial community, enriched by *Proteobacteria* and depleted by *Firmicutes* and *Bacteroidetes*. Pro-inflammatory genera, including *Stenotrophomonas* (e.g., *S. maltophilia*) and *Chryseobacterium* (e.g., *C. indologenes*), were enriched predominantly in males. Collectively, these findings identified NLRP12 inflammasome-associated signaling and sex-dependent microbial dysbiosis as key mechanisms underlying male-biased lung inflammation, informing sex-specific risk assessment of PFAS alternatives.

## 1 Introduction

Per and polyfluoroalkyl substances (PFAS) are man-made, surface-active chemicals used for their chemical stability. Perfluorooctane sulfonate (PFOS) is one of the most common PFAS and was added to the Stockholm Convention in 2009 because of its persistence, bioaccumulation, and multi-organ toxicity (1). The main substitute, 6:2-chlorinated polyfluoroalkyl ether sulfonic acid (6:2 Cl-PFESA, also referred to as F-53B), was developed as a functional equivalent mist suppressant and has since been manufactured and used on an industrial scale in the Chinese electroplating industry (2). The substitution, however, has not delivered the intended reduction in hazard. The terminal chlorine atom and the ether linkage of 6:2 Cl-PFESA provide environmental stability that is at least as great as PFOS, and an estimated human elimination half-life of approximately 15.3 years is greater than the replacement compound (3). As a result, researchers have found it in various environmental media including industrial wastewater (43-112μg/L), river water (2ng/L-7.6μg/L)ambient air (nd-1.12pg/m^3^) and municipal sludge (0.02–209 ng/g) (4). Thus, 6:2 Cl-PFESA illustrates ‘regrettable substitution’, which means that the regulatory stress on the legacy chemical does not reduce the exposure burden, but rather it shifts it to the substitute.

This ubiquity in the environment is reflected in human exposure as well: 6:2 Cl-PFESA has been found in breast milk, urine and serum (5). In the general population, the range of serum concentrations is 0.22-9.12 ng/mL, while in electroplating operators, the values are much higher (0.42–941 ng/mL), which accounts for 8.69-28.0% of the total concentration of PFAs in the blood samples (6). Since then, experimental studies have confirmed immunotoxicity, hepatotoxicity, reproductive and endocrine disruption as characteristic features of 6:2 Cl-PFESA toxicity (7). Yet this literature has been organized almost exclusively around the liver, the gut, and the reproductive axis. The respiratory system has remained essentially unexamined, despite the fact that the alveolar epithelium is continuously perfused, immunologically reactive, and therefore directly accessible to systemically distributed xenobiotics.

The omission is difficult to justify because the lung is emerging as a bona fide target of PFAS toxicity. Epidemiological studies have linked PFAS exposure to risk of chronic obstructive pulmonary disease (COPD) and impaired lung function (8–10). Mechanistically, PFAS exert pro-inflammatory and immunomodulatory effects through peroxisome proliferator-activated receptor (PPAR) activation, sex-hormone signaling disruption, NF-κB and JAK-STAT modulation, epithelial barrier impairment, and oxidative stress-driven tissue injury (11–13). It remains untested whether 6:2 Cl-PFESA activates these pathways in the lung. In general, the innate immune sensing mechanism that integrates chemical stress and triggers self-sustaining pulmonary inflammation, particularly the nucleotide-binding oligomerization domain (NOD)-like receptor (NLR) family, which is responsible for priming and assembling inflammasomes within airway and alveolar epithelium, has not been examined for PFAS alternatives. This means that there is a missing link between the documented exposure and the molecular events that might actually turn that exposure into a respiratory disease.

In addition to the host molecular pathways, the lung also has a diverse microbial community with a growing role in maintaining mucosal balance, controlling immune responses, and modulating susceptibility to respiratory diseases (14). The pulmonary microbiome interacts closely with the host immune system, and its dysbiosis has been implicated in cystic fibrosis, asthma, COVID-19 infection, pneumonia, Chronic obstructive pulmonary disease (COPD) (15). Critically, the two axes are not independent; rather, they are linked through a mechanistic cascade in which dysbiosis promotes Gram-negative bacterial expansion, increasing pulmonary LPS accumulation, which in turn primes NLR inflammasome activation and drives inflammatory signaling. Gram-negative expansion within a dysbiotic airway community provides a sustained source of lipopolysaccharide (LPS), the canonical priming signal for NLR-dependent inflammasome activation, so that a chemically induced shift in microbial composition can be transduced into persistent innate immune activation. A chemical-microbiota-inflammasome axis therefore offers a testable mechanistic bridge between systemic 6:2 Cl-PFESA exposure and localized pulmonary injury. Whether 6:2 Cl-PFESA perturbs the lung microbiome at all, however, remains entirely unknown.

A final and frequently neglected determinant is sex. Animal studies demonstrate that male and female rodents exhibit differential susceptibility to PFAS-induced hepatotoxicity and immunotoxicity. In a 14-day rat study, acute oral exposure of 6:2 Cl-PFESA caused multi-organ toxicity with sex-dependent mechanisms (16), and in zebrafish, it caused liver-dominant, sex-specific lipid depletion and PPAR-mediated metabolic disruption (17). Because PFAS toxicokinetic, redox buffering capacity, and immune reactivity are each sex-dependent, sex is likely to act as a determinant of pulmonary risk rather than as a nuisance covariate. Nevertheless, no study has resolved sex-specific pulmonary responses to 6:2 Cl-PFESA, nor established whether host transcriptional and microbial responses diverge in parallel or in opposition.

In this study, we tested the hypothesis that subchronic 6:2 Cl-PFESA exposure produces sex-dependent lung inflammation by coupling epithelial redox stress with NLR-dependent inflammatory signaling and lung microbiota dysbiosis. We comprehensively evaluated lung inflammation of 6:2 Cl-PFESA in male and female mice by integrating histopathological assessment, transcriptomic profiling, *in vitro* mechanistic studies, and lung microbiome analysis. We aimed to (1) characterize the sex-dependent pulmonary and systemic effects of subchronic 6:2 Cl-PFESA exposure; (2) identify key molecular pathways mediating the inflammatory response; (3) determine whether 6:2 Cl-PFESA induces lung microbiota dysbiosis and its association with host inflammatory gene expression; and (4) establish an integrated framework linking host responses, microbial alterations, and sex-dependent susceptibility. Our findings revealed that 6:2 Cl-PFESA elicits sex-dependent lung inflammation through NLRP12 inflammasome activation and concurrent microbial dysbiosis, providing critical insights for sex-specific risk assessment of PFAS alternatives.

## 2 Materials and experimental design

### 2.1 Dose optimization study

A preliminary pilot study was first conducted on male C57BL/6 mice (6 weeks old; 18–20 g; Hubei Experimental Animal Inspection Station, Wuhan, China; SYXK(Hubei) 2020-0084) to determine the optimum dosage for the mechanistic study. Stock solutions were prepared by dissolving 6:2 Cl-PFESA (99% purity, CAS: 73606-19-6; NONA Chemical, Xiangyang, China) in 0.2% Tween 20. Mice were randomly assigned to receive 0.5, 5, or 10 mg/kg body weight (BW) of 6:2 Cl-PFESA or vehicle control (0.2% Tween 20) via oral gavage for 28 days (n=5/group). Endpoints included weekly body weight monitoring, lung histopathological evaluation via H&E staining, and serum levels of inflammatory cytokines (e.g., IL-1β, TNF-α, IL-6). Based on the pilot results, the 10 mg/kg/BW dose was selected as the optimal dose for subsequent mechanistic studies, as it induced the most pronounced phenotypic changes without overt toxicity, consistent with previously reported PFAS toxicity studies (18, 19).

### 2.2 Main mechanistic study

Based on the pilot findings, a separate cohort of C57BL/6 mice (6 weeks old; males 18–20 g, females 16–18 g; Hubei Experimental Animal Inspection Station, Wuhan, China, License No. SCXK(E)20200019) were assigned to four experimental groups (n=10/sex/group): (1) Control Male (vehicle), (2) Control Female (vehicle), (3) PFESA Male (10 mg/kg/BW), and (4) PFESA Female (10 mg/kg/BW). The working dose of 10 mg/kg/BW was freshly prepared daily by further diluting the stock solution with 0.2% Tween 20. Mice were administered the compound or vehicle control via oral gavage once daily for 28 consecutive days. All animals were housed under standard conditions (22 ± 3 °C, 40-60% humidity, 12-h light-dark cycle) at the National Veterinary Drug Residue References Laboratory, Huazhong Agricultural University, Wuhan, China. Mice had unrestricted access to SPF diet and water, and body weights were recorded weekly.

### 2.3 Sample collection

At the end of the 28-day exposure period, all mice were euthanized by gradual CO_2_ asphyxiation, followed by cervical dislocation to ensure death. Whole blood was allowed to clot at room temperature for 30 min before centrifugation (3,000 rpm, 10 min, 4 °C). Serum was carefully aspirated and preserved at -80 °C. Sterile Eppendorf tubes were used to collect and place lung tissue prior to microbiome and transcriptome analysis.

### 2.4 Histopathology

Lung tissues were fixed in 4% paraformaldehyde, embedded in paraffin and then cut into thin sections (3-5 μm). Sections were deparaffinized, rehydrated and stained with hematoxylin and eosin (H&E). Following dehydration and mounting, histological evaluation was performed under a light microscope.

### 2.5 Measurements of inflammatory cytokines, lipopolysaccharide and oxidative stress markers

Serum concentrations of IL-1β (JM-02323M1), IL-6 (JM-02446M1), TNF-α (JM-02415M1), IL-4 (JM-02448M1), and IL-10 (JM-02459M1) were assessed using kits from Jingmei Biological Technology Co., Ltd, China, and lung tissue LPS was measured with a commercially available kit (MM-92600101, Jiangsu Miemian Industrial Co., Ltd), following the manufacturer’s protocols. For biochemical analysis, lung tissues were homogenized in PBS (1:9, w/v) using a tissue homogenizer (L-HOB-MINI, Beijing Lajieke Technology Co., Ltd.), followed by centrifugation (3000 rpm, 10 min, 4 °C). The supernatants obtained were analyzed to determine the levels of catalase (CAT, A007-1-1), glutathione (GSH, A006-2-1), malondialdehyde (MDA, A003-1-2), and superoxide dismutase (SOD, A001-1-2) using kits from Nanjing Jiancheng Biotechnology, Nanjing, China. Bicinchoninic Acid (BCA) assay kit (Spark Jade, EC0001-A) was used to determine total protein content, and all biochemical data were normalized to total protein content.

### 2.6 Transcriptome analysis

RNA extraction was performed consistently and efficiently by automated systems (VNP-32P and VNP-96P, Vazyme, Nanjing, China). Standard protocols were used to construct the Poly(A)-selected libraries, which were then sequenced on an Illumina NovaSeq X Plus platform. The mouse reference genome (GRCm39) and the corresponding gene annotations were retrieved from public genome databases. High-quality reads were obtained by trimming with Trim Galore and filtered reads were aligned to the GRCm39 assembly with STAR v2.7.11a. Resulting alignments were converted and processed with SAMtools v1.18, and featureCounts v2.0.6 was used to count genes, which were input into R for differential expression analysis using DESeq2; genes with a Benjamini-Hochberg adjusted *p*-value<0.05 and absolute log_2_fold change ≥1 were considered significant. Data visualization (heatmaps and volcano plots) was carried out using ClusterGVis and ggplot2. The functional enrichment analysis of the KEGG pathway was evaluated using the ShinyGO v0.82.

### 2.7 Cell culture and treatment

Human type II alveolar epithelial cells (A549) were obtained from Warner Bio (WN-10176, Wuhan, China) and cultured in Ham’s F12 medium (Shanghai Yuanpei Biotechnology Co., Ltd.), supplemented with 1% penicillin/streptomycin (Gibco) and 10% fetal bovine serum (SERANA). A549 cells are commonly utilized as an *in vitro* surrogate for human type II alveolar epithelial cells (AECII). Prior studies reported that these cells display relevant antigen-presenting surface molecules and biochemical and metabolic features comparable to those of freshly isolated human AEC II under both resting and stimulated conditions (20–23). Here, this cell model was used specifically for epithelial cell-autonomous biochemical and signaling validation of proposed mechanisms.

Human THP-1 monocytic cells were purchased from Warner Bio (Wuhan) Co., Ltd. and maintained in RPMI 1640 medium (Gibco) supplemented with 10% fetal bovine serum (SERANA) and 1% penicillin/streptomycin (Gibco). Cells were cultured in a humidified incubator at 37 °C with 5% CO_2_. To induce differentiation into macrophage-like cells, THP-1 monocytes were treated with 100 ng/mL phorbol 12-myristate 13-acetate (PMA) for 24 h. Following differentiation, the adherent THP-1-derived macrophage-like cells were used for subsequent exposure and mechanistic analyses.

For exposure experiments, A549 cells (0.35 × 10^6^ cells/well) were seeded in 6-well plates and incubated overnight at 37 °C and 5% CO_2_. A549 cells and differentiated THP-1 cells were subsequently exposed to 6:2 Cl-PFESA at concentrations of 5 and 10 μM for 48 h. During both exposure regimens, the exposure medium was refreshed every 24 h. The selection of exposure concentrations was based on relevant literature (24, 25) and validated by CCK-8 cell viability assays. The stock solutions of 6:2 Cl-PFESA were prepared in DMSO (20220314, Sinopharm Group) and diluted with the corresponding culture medium to obtain working solutions. The final DMSO concentration in both the control and exposure groups was controlled at 0.1%. To improve translational interpretation between *in vivo* and *in vitro* exposure regimens, the *in vitro* doses (5 and 10 µM) were also expressed as moles per cell, as previously recommended (26). Based on a seeding density of 0.35 × 10^6^ cells per well and a culture volume of 2 mL, the 5 μM and 10 μM 6:2 Cl-PFESA exposures correspond to approximately 2 × 10^-^¹^4^ mol/cell (20 pmol per 10^6^ cells) and 4 × 10^-^¹^4^ mol/cell (40 pmol per 10^6^ cells), respectively.

### 2.8 Cell viability assay

The Cell Counting Kit-8 (CCk-8; Beijing Lanjike Technology Co., Ltd., Beijing, China) was used to check the cell viability. Cells were seeded in 96-well plates at 3,000 cells/well, cultured for 24 h, and then exposed to 6:2 Cl-PFESA (0.075-40 μM) for 24 or 48 h. CCK-8 reagent (10 μL/well) was added and the plates were incubated for 2 h at 37 °C. Absorbance was measured at 450 nm, and viability was calculated according to the manufacturer’s given formula. Three independent experiments were performed with five technical replicates per condition.

### 2.9 Detection of intracellular ROS

Fluorescent probe, 2′,7′-dichlorodihydrofluorescein diacetate (DCFH-DA) was used to measure intracellular ROS. In brief, Cells were seeded in a 6-well plate and incubated overnight. Following incubation. Cells were exposed to 6:2 Cl-PFESA (5 and 10 μM) for 48 h. The control group was treated with the same amount of vehicle (0.1% DMSO) as the test groups, but no test compound. After exposure, the cells were washed twice with PBS and the culture medium was removed. A working solution of DCFH-DA was prepared by 1000-fold dilution in PBS, and 1 mL of probe solution was added per well. Plates were incubated for 30 min in the dark at 37 °C. The solution containing DCFH-DA was removed after incubation and washed again with PBS. Later, 1 mL of PBS was added to each well, and the fluorescence was immediately observed under light-proof conditions. Intracellular ROS was visualized and measured by a Fluorescence Inverted Microscope (IX73, Olympus).

### 2.10 Caspase-1 activity assay

Caspase-1 activity was measured in A549 and THP-1 cells treated with vehicle control, 5 μM, or 10 μM 6:2 Cl-PFESA using a commercial Caspase-1 Activity Assay Kit (Colorimetric Method; E-CK-A381, Elabscience Biotechnology Co., Ltd.) according to the manufacturer’s instructions. Briefly, cell lysates were prepared using the supplied Cell Lysis Buffer and centrifuged at 11,000 × g for 10–15 min at 4 °C. Protein concentrations were determined using the Bradford method. Equal amounts of protein extracts were incubated with 2× Reaction Buffer and Ac-YVAD-pNA substrate at 37 °C for 1–2 h. Absorbance was measured at 405 nm using a microplate reader. Caspase-1 activity was calculated using a pNA standard curve, normalized to total protein concentration, and expressed as U/mg protein.

### 2.11 Targeted gene knockdown

To investigate the functional contribution of *NLRP12* to 6:2 Cl-PFESA-induced inflammatory responses, A549 and THP-1 cells were transfected with two independent *NLRP12*-targeting siRNAs (siNLRP12–1 and siNLRP12-2) or non-targeting control siRNA (siNC) synthesized by Beijing Tsingke Biotech Co., Ltd. using JetPRIME^®^ transfection reagent (0000004416, Polyplus-transfection, France) according to the manufacturer’s instructions. Briefly, 0.5 × 10^6^ A549 cells were seeded into six-well plates and transfected after 24 h. Following 6 h of transfection, the medium was replaced with fresh complete medium, and knockdown efficiency was evaluated by qRT-PCR and Western blotting. The siRNA sequence with higher silencing efficiency (siNLRP12-1) was selected for subsequent functional experiments (**Supplementary Table S1**).

The initial knockdown experiment was performed by exposing siNC- and siNLRP12-1-transfected A549 and THP-1 cells to 10 μM 6:2 Cl-PFESA for 48 h to determine whether *NLRP12* silencing attenuated 6:2 Cl-PFESA-induced inflammatory responses. Subsequently, a 2 × 2 factorial experimental design was conducted to distinguish the effects of *NLRP12* depletion and 6:2 Cl-PFESA exposure, including four groups: siNC + DMSO, siNC + 10 μM 6:2 Cl-PFESA, siNLRP12 + DMSO, and siNLRP12 + 10 μM 6:2 Cl-PFESA. Following transfection, cells were exposed to 6:2 Cl-PFESA or vehicle control for 48 h, and inflammatory markers were evaluated.

### 2.12 Stenotrophomonas maltophilia exposure

*Stenotrophomonas maltophilia (S. maltophilia)* (ATCC 17666; BNCC363070) was obtained from the BeNa Culture Collection (BNCC, China) and recovered under aerobic conditions according to the supplier’s recommendations. For bacterial challenge experiments, *S. maltophilia* was cultured to the logarithmic growth phase, harvested by centrifugation at 5,000 × *g* for 10 min, washed twice with sterile PBS, and resuspended in antibiotic-free DMEM/F-12 medium at 1 × 10^7^ CFU/mL. The bacterial concentration was verified by serial dilution and colony counting.

A549 cells were pre-exposed to 10 μM 6:2 Cl-PFESA for 48 h in antibiotic-free medium, with 0.1% DMSO serving as the vehicle control. Following PFESA pre-exposure, *S. maltophilia*, prepared independently in the absence of PFESA, was added to the designated groups at a multiplicity of infection (MOI) of 100:1 and incubated for 6 h at 37 °C in 5% CO_2_. Four experimental groups were established: control (0.1% DMSO), *S. maltophilia* alone (SMA), PFESA alone, and PFESA pre-exposure followed by *S. maltophilia* challenge (PFESA + SMA). Following the 6-h bacterial challenge, cells were washed three times with sterile PBS and incubated with gentamicin (600 μg/mL) for 1 h at 37 °C to eliminate extracellular bacteria. Cells were then washed with ice-cold PBS and harvested for total RNA extraction followed by qPCR.

### 2.13 RNA extraction and qRT-PCR

Cells were subjected to total RNA extraction using the VeZol reagent (Vazyme Biotech Nanjing, R411-02-AA). The NanoDrop 2000 spectrophotometer (Thermo Fisher Scientific, MA, USA) was used to assess the RNA concentration and purity. HiScript II Q RT SuperMix with gDNA wiper (R223-01, Vazyme) was used to synthesise cDNA. The targeted genes were amplified by qRT-PCR with primer pairs listed in **Supplementary Table S2** and a SYBR Green qPCR master mix (Vazyme) on a Bio-Rad CFX Connect real-time PCR system (Bio-Rad, CA, USA). β-actin was the internal control, and the 2^^−ΔΔCt^ method was employed to determine the relative expression of mRNA.

### 2.14 Western blot analysis

Protein samples were extracted using RIPA buffer containing PMSF (Beyotime) and quantified by BCA assay (P0010, Beyotime). To separate proteins, 6-8 μL of the supernatant was subjected to SDS-PAGE and transferred to a polyvinylidene fluoride (PVDF) membrane (0000432953, Merck Millipore Ltd). The membranes were blocked with 5% skim milk and probed with primary antibodies overnight and HRP-conjugated secondary antibodies for 2 h. Rewashed with TBST and visualized using ECL chemiluminescence substrate kit (BL520B, Biosharp) with imaging system ‘Tiana’. The list of antibodies used is given in **Supplementary Table S3**.

### 2.15 16S rRNA sequencing

Lung tissues from C57BL/6 mice were sampled aseptically, immediately frozen in liquid nitrogen, and stored at -80 °C. Six lung samples per group (control and 10 mg/kg BW 6:2 Cl-PFEAS-treated; males and females) were processed for 16S rRNA gene sequencing (Metware Biotechnology Co., Ltd., Wuhan, China). Genomic DNA was extracted, and the V3-V4 regions were amplified using primers: 341F (CCTATGGGRGCTCGGAGGCAG) and 806R (GGACTACHVGGGTATCTAAT) on a GeneAmp 9700 thermal cycler. Electrophoresis on a 2% agarose gel was used to confirm the PCR products, subsequently purified with the Qiagen gel extraction kit. Sequencing libraries were prepared using the TruSeq DNA PCR-Free kit (Illumina). Library quantification was conducted using Qubit and qPCR, and sequenced on an Illumina NovaSeq 6000 (PE250). Raw amplicon reads were denoised with Deblur to remove low-quality, short, and chimeric sequences, and amplicon sequence variants (ASVs) were generated for taxonomic assignment. After per-sample normalization, alpha diversity indices (Shannon, Simpson, Chao1, ACE, Observed ASV) were calculated in QIIME, and beta diversity was assessed by PCoA, and NMDS. Between-group comparisons at each taxonomic level were performed using Metastats in R with permutation testing; the Benjamini-Hochberg false discovery rate (FDR) method was applied to adjust *p*-values to q-values.

### 2.16 Statistical analyses

Statistical analyses were performed with GraphPad Prism 9.0 (GraphPad Software, CA, USA) and all data are reported as mean ± standard deviation (SD). A t-test was used to compare two groups while for multiple groups comparison, one-way or two-way ANOVA was used followed by Tukey’s *post hoc* test. A p-value below 0.05 was considered statistically significant.

## 3 Results

### 3.1 Lung toxicity of 6:2 Cl-PFESA in mice

In the pilot dose-range finding study, administration of the compound at 0.5 and 5 mg/kg produced no significant changes in body weight, circulating inflammatory cytokines (IL-1β, TNF-α, IL-6) or tissue histopathology relative to vehicle controls. In contrast, the 10 mg/kg body weight dose induced marked alterations in all of these parameters (**Supplementary Figures S1A–E**) and was therefore selected for the main mechanistic cohort. To investigate whether 6:2 Cl-PFESA exposure elicits differential responses, we next monitored body weight changes and evaluated pulmonary histopathology in both male and female mice. As shown in **Figures 1A, B**, Males exhibited an earlier onset of 6:2 Cl-PFESA-induced body weight loss compared to females, with significant weight reduction observed from day 7 in males and from day 14 in females. However, by day 28, the magnitude of weight loss was comparable between sexes, with both PFESA-exposed groups showing significant weight reduction relative to their respective controls (*p* < 0.0001 for both), while no significant difference was observed between PFESA-exposed males and females (*p* > 0.05). In addition, lung tissue histological examination revealed evidence of significant injury in the exposed male with vascular congestion, collapsed alveoli, thickened septa, emphysematous alterations, and dense fibrillar infiltration. This was more pronounced in males (**Figure 1C**). Serum cytokine concentrations were measured to further explore the systemic effects. Exposure resulted in a significant rise in proinflammatory cytokines IL-1β, IL-6, and TNF-α (**Figures 1D–F**) as well as a significant decrease in anti-inflammatory cytokines IL-4 and IL-10 (**Figures 1G, H**) (p< 0.05). In particular, the elevations in IL-1β and IL-6, and the reduction in IL-4 were greater in males. Furthermore, LPS in lung tissue homogenate was significantly elevated (p < 0.05) after exposure in both sexes, and higher levels were seen in males (**Figure 1I**), suggesting that the male lungs presented a higher endotoxin load. These results indicate significant sex-specific histological and inflammatory alterations caused by 6:2 Cl-PFESA, with males being more susceptible.

**Figure 1:**
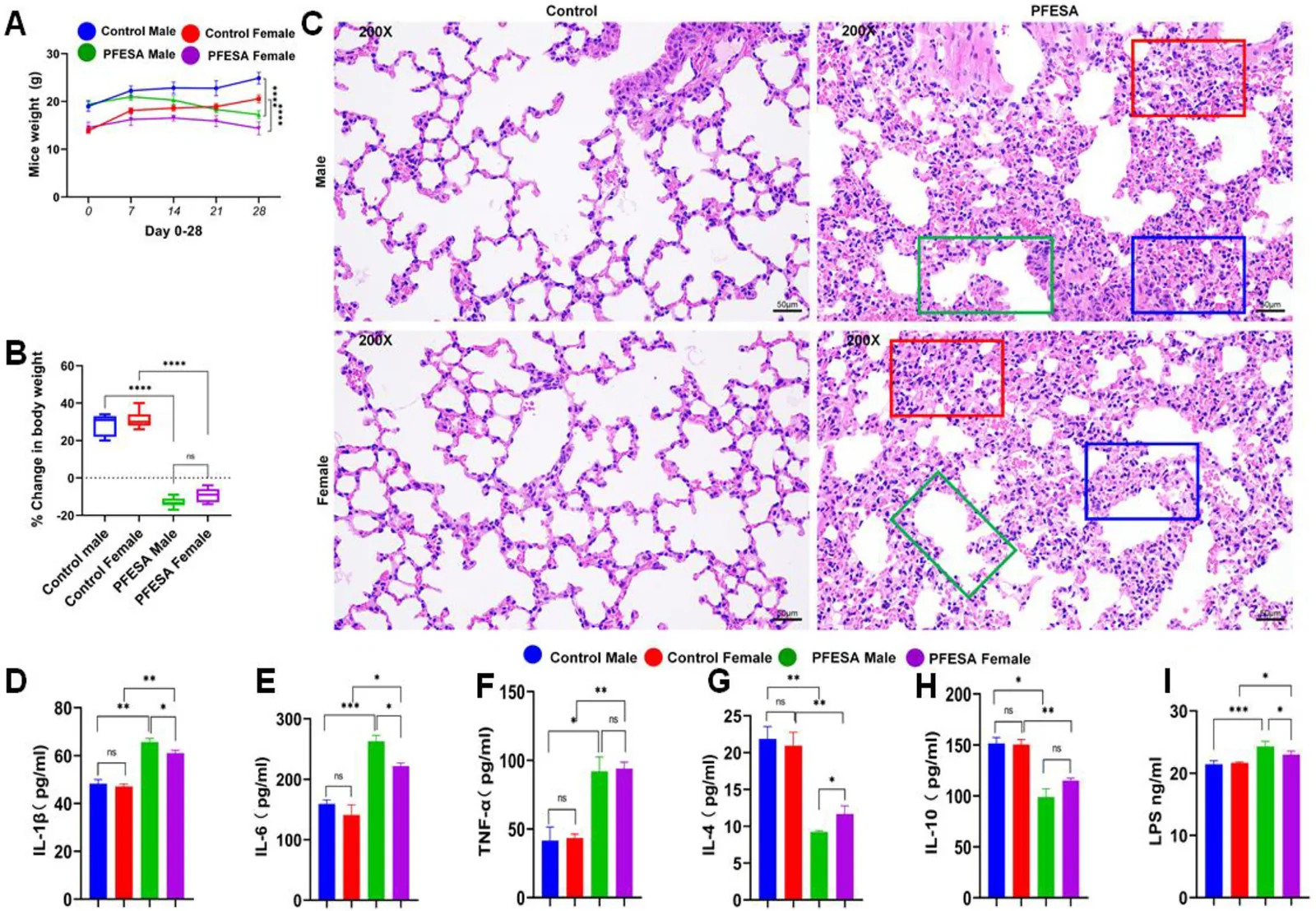
Sex-dependent effects of 6:2 Cl-PFESA exposure on body weight, lung histology, inflammatory cytokines, and LPS level in mice. **(A)** Body weight loss in mice following exposure. **(B)** The percent change in body weight, showing greater loss in exposed males. (n = 10 independent biological replicates per group) **(C)** Representative H&E staining images from lung sections of male (top) and female (bottom) mice (200x magnification). The red box indicates inflammatory cell infiltration, the blue box indicates alveolar septal fibrosis, and the green box indicates emphysema. (n = 3 independent biological replicates per group) **(D–H)** Levels of IL-1β, IL-6, TNF-α, IL-4, and IL-10. (n = 3 independent biological replicates per group) **(I)** Lung homogenate LPS concentrations. (n = 3 independent biological replicates per group). Data were analyzed by two-way ANOVA, represented as mean ± S.D. ns, non-significant; *p < 0.05; **p < 0.01; ***p < 0.001; ****p < 0.0001).

### 3.2 6:2 Cl-PFESA induces oxidative stress and activates MAPK signaling

#### 3.2.1 6:2 Cl-PFESA elicits oxidative stress markers in mouse lung

To evaluate oxidative damage *in vivo*, biomarkers were analyzed in mouse lung tissue. Exposure to 6:2 Cl-PFESA 10 mg/kg/BW significantly elevated MDA, CAT, and GSH levels in both sexes (**Figures 2A–C**), indicating enhanced oxidative stress. Notably, the increases in MDA and CAT were significantly greater in males than in females (**Figures 2A, B**). Conversely, SOD activity was significantly decreased, with a greater reduction observed in males (**Figure 2D**).

**Figure 2:**
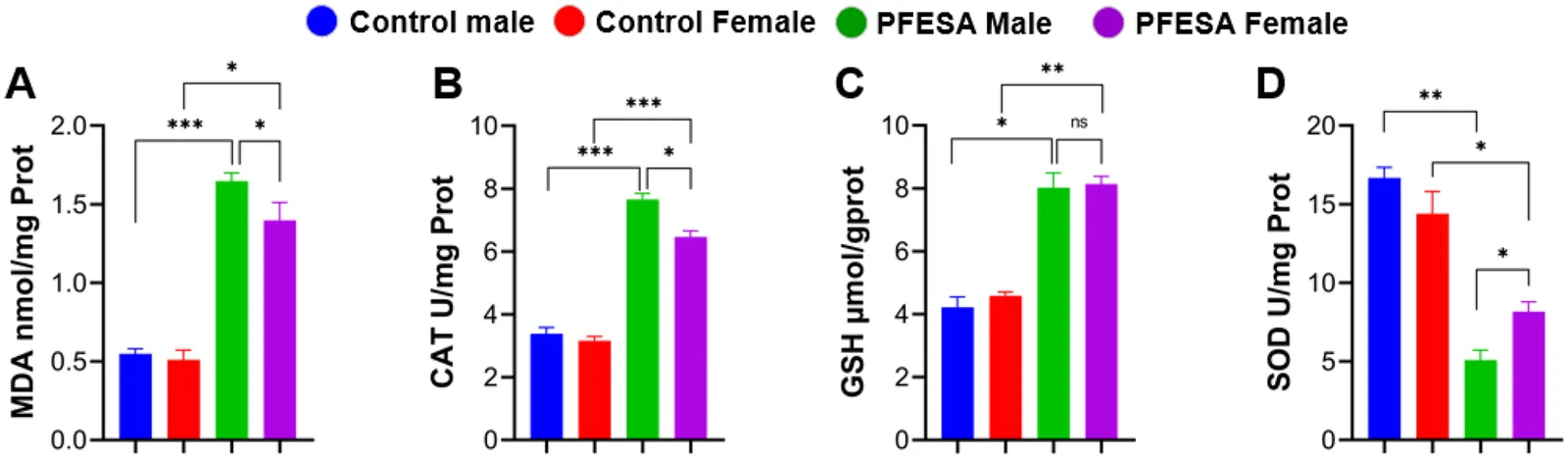
Exposure to 6:2 Cl-PFESA induced oxidative stress in male and female mice. **(A–C)** MDA, CAT, and GSH levels in exposed mice. **(D)** SOD activity. (n = 3 independent biological replicates per group). Data were analyzed by two-way ANOVA and presented as mean ± S.D. ns, non-significant; *P < 0.05; **P < 0.01; ***P < 0.001.

#### 3.2.2 6:2 Cl-PFESA reduced cell viability

To evaluate the cellular effects of 6:2 Cl-PFESA, cell viability was assessed in A549 and THP-1 cells following exposure to increasing concentrations of the compound for 24 and 48 h. In both cell types, 6:2 Cl-PFESA induced time- and concentration-dependent reductions in cell viability. No significant changes in viability were observed after 24 h across the tested concentration range in either A549 or THP-1 cells (**Supplementary Figures S2A, C**). In contrast, after 48 h of exposure, A549 cell viability was significantly reduced at 10 μM and further decreased with increasing concentrations, reaching approximately 50% of the control at 40 μM (**Supplementary Figure S2B**). Similarly, THP-1 cell viability was significantly reduced at 10 μM, with more pronounced decreases at 20 and 40 μM, reaching approximately 65% of the control at 40 μM (**Supplementary Figure S2D**). These findings demonstrate that the effects of 6:2 Cl-PFESA on cell viability are dependent on both exposure duration and concentration, with a greater reduction observed at the highest concentration tested. To minimize potential confounding effects associated with reduced cell viability, 5 and 10 μM 6:2 Cl-PFESA were selected for subsequent mechanistic studies.

#### 3.2.3 6:2 Cl-PFESA induces intracellular ROS production and activates MAPK signaling

To further validate whether the oxidative stress observed *in vivo* was recapitulated at the cellular level and to investigate the underlying signaling mechanisms, complementary *in vitro* experiments were performed using A549 and THP-1 cells. Exposure to 6:2 Cl-PFESA significantly increased intracellular ROS levels in both cell types in a concentration-dependent manner (**Figures 3A–D**), confirming enhanced oxidative stress. Consistent with this response, transcriptional analysis revealed differential regulation of MAPK-related genes (**Figures 3E, F**). In both A549 and THP-1 cells, *p38* expression was significantly downregulated, whereas *ERK* and *JNK* were significantly upregulated following 6:2 Cl-PFESA exposure. The downstream stress-responsive genes *HIF-1*, *Nrf2*, and *VEGFA* were also significantly upregulated in both cell types, with generally stronger responses at 10 μM.

**Figure 3:**
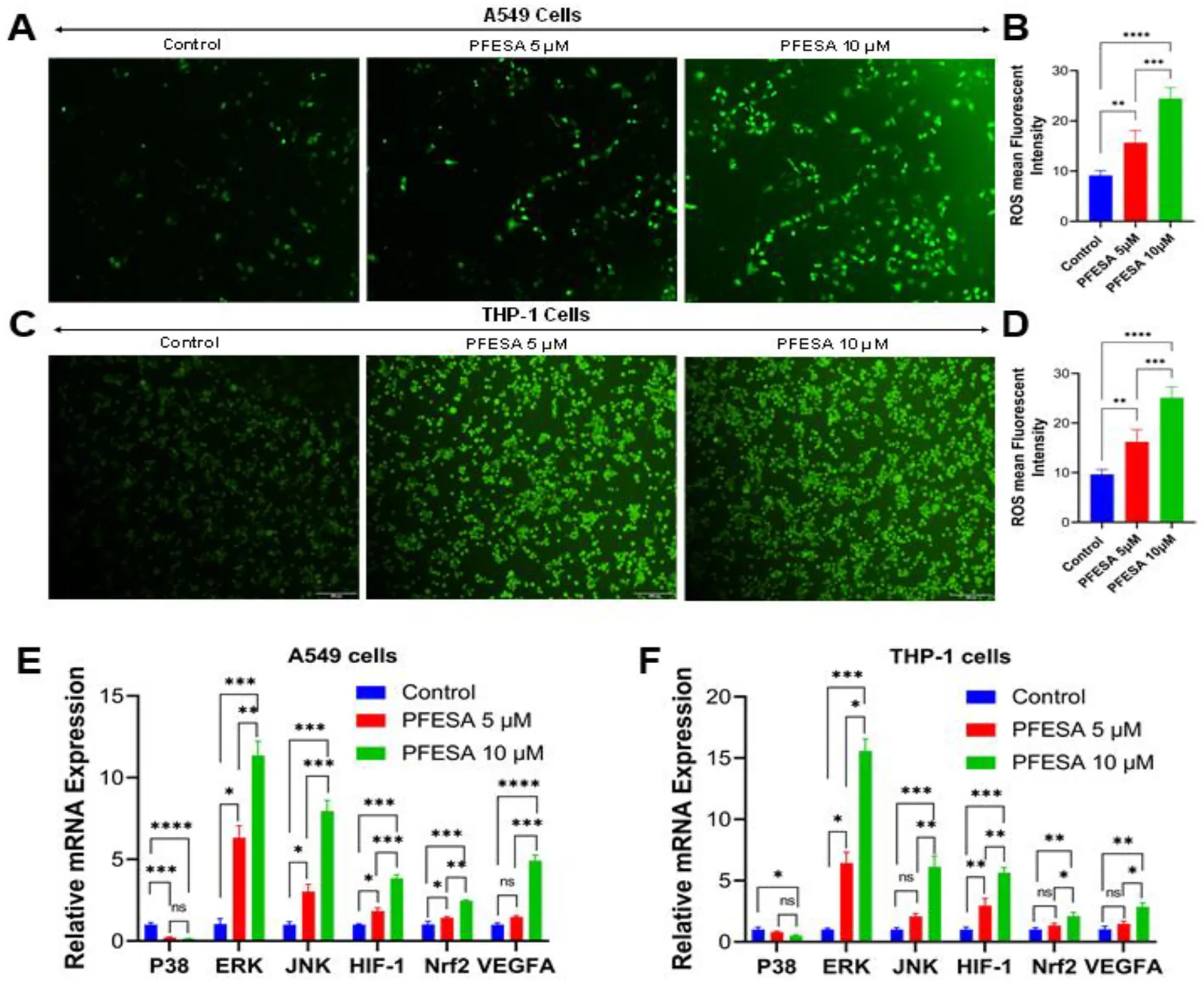
Elevation of ROS and changes in oxidative stress-related genes in A549 and THP-1 cells following exposure to 6:2 Cl-PFESA. **(A–D)** Representative fluorescence images and quantification of intracellular ROS accumulation in A549 and THP-1 cells. **(E, F)** Relative mRNA expression of *p38, ERK, JNK, HIF-1, Nrf2*, and *VEGFA* in A549 and THP-1 cells, respectively. Data are presented as mean ± S.D. (n = 3 independent biological replicates per group) and were analyzed by two-way ANOVA. ns, non-significant; *p* < 0.05; *p < 0.01; **p < 0.001; ***p < 0.0001.

Collectively, these findings demonstrate that 6:2 Cl-PFESA induces ROS accumulation and differential regulation of MAPK-associated genes in both alveolar epithelial and monocytic cells, accompanied by increased expression of downstream oxidative stress- and hypoxia-responsive genes.

### 3.4 6:2 Cl-PFESA reprograms the pulmonary transcriptome

Next, we performed RNA-Seq analysis of mouse lung tissue following exposure to 6:2 Cl-PFESA 10 mg/kg/body weight to investigate the mechanisms behind 6:2 Cl-PFESA-induced lung damage. Detailed quality control and mapping data are presented in **Supplementary Table S4**. Differentially expressed genes (DEGs) were defined as those with |log_2_ (fold change) | ≥ 1 and an adjusted p-value < 0.05.

In male mice, 88 DEGs were identified following 6:2 Cl-PFESA exposure, of which 51 were downregulated, and 37 were upregulated (**Figure 4B**). In females, a substantially larger number of DEGs (209 genes) were detected, comprising 87 DEGs downregulated and 122 upregulated (**Figure 4C**). As illustrated in the Venn diagram (**Figure 4A**), the overlap between sexes was remarkably limited: only 21 genes (7.6%) were commonly regulated in both male and female mice, whereas 188 genes (68.1%) were uniquely altered in females and 67 genes (24.3%) were unique to males. These findings highlight a sex-specific transcriptional response to 6:2 Cl-PFESA exposure, with female lungs showing a greater overall gene expression alteration and a predominance of upregulated genes.

**Figure 4:**
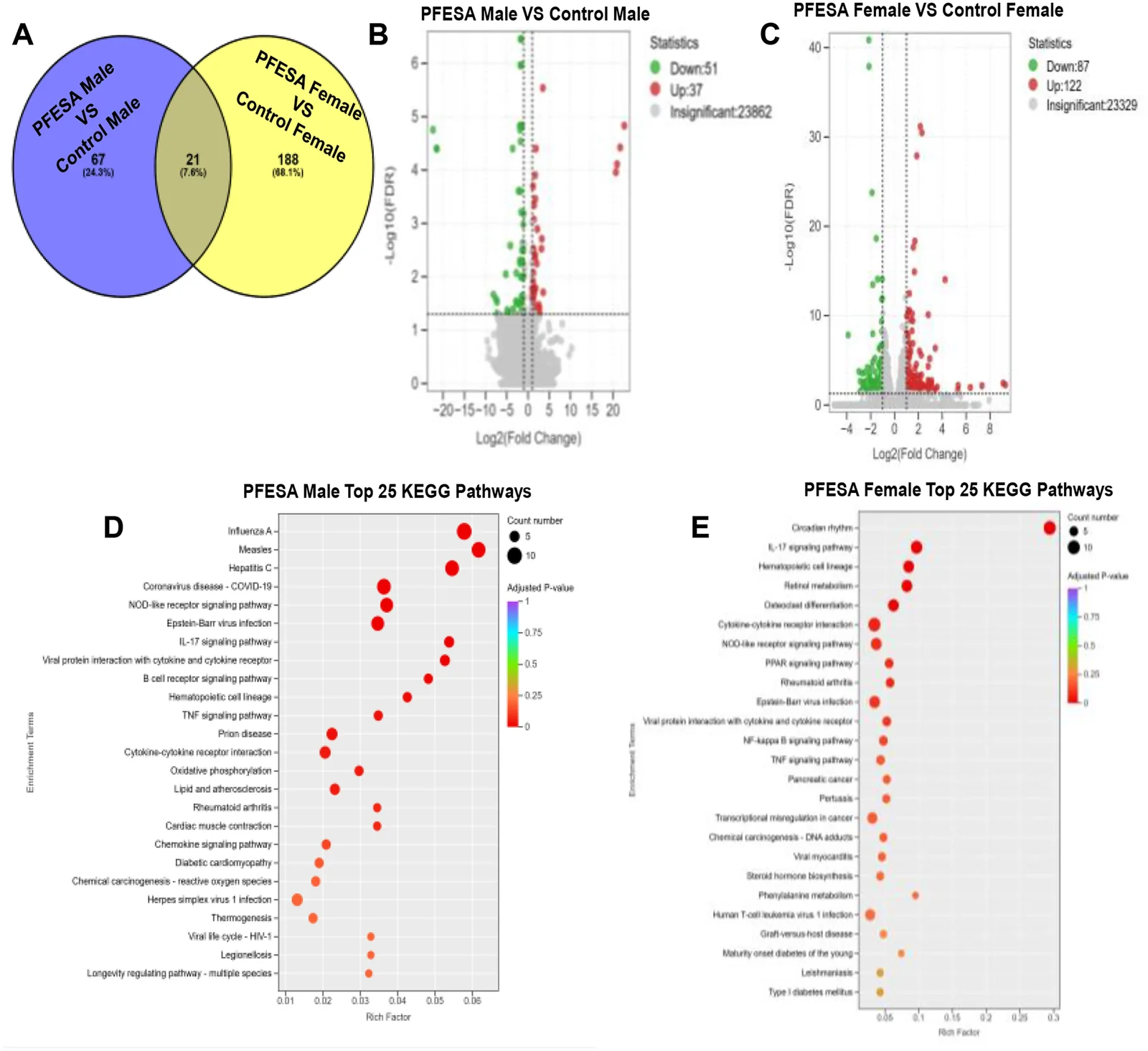
6:2 Cl-PFESA exposure changes gene expression profiling in mouse lungs (n = 3 independent biological replicates per group). **(A)** The Venn diagram highlights shared and core DEGs between the control and exposed groups. **(B, C)** Volcano plots display male and female DEGs, respectively. **(D, E)** Up- and downregulated genes in KEGG pathways of male and female mouse lung tissue, respectively. The color indicates the p-adj value, and the circle size shows the number of genes.

Furthermore, we performed KEGG pathway analysis (adjusted *p*-value<0.05) on both up- and downregulated DEGs (**Supplementary Tables S5, S6**). KEGG pathway analysis (**Figures 4D, E**) revealed enrichment of several inflammatory pathways in both sexes, including the NOD-like receptor signaling pathway, IL-17 signaling, and TNF signaling (**Figures 4D, E**). Among these, the NOD-like receptor signaling pathway was significantly enriched in both the control and exposed comparison groups, with *p*-values of 2.8 × 10^^-5^ and 0.003, respectively. The gene *NLRP12* was upregulated in both male and female groups, while *IRF7* was downregulated only in males and *Bcl2l1* only in females.

### 3.5 6:2 Cl-PFESA activates NLRP12-driven inflammatory cascade

#### 3.5.1 Dose-dependent induction of inflammatory responses *in vitro*

To determine whether 6:2 Cl-PFESA induces NLRP12-associated inflammasome activation, A549 and THP-1 cells were exposed to 5 and 10 μM 6:2 Cl-PFESA, followed by assessment of inflammasome-related transcriptional and protein changes and functional activation markers.

qRT-PCR analysis showed that 6:2 Cl-PFESA increased the mRNA expression of *NLRP12, IL-1β, IL-6, TNF-α*, and *caspase-1* in both A549 and THP-1 cells, with generally greater responses at 10 μM. In contrast, *IRF7* and *Bcl2l1* expression was significantly reduced in both cell types (**Figures 5A, D**), indicating a shift towards a pro-inflammatory transcriptional profile.

**Figure 5:**
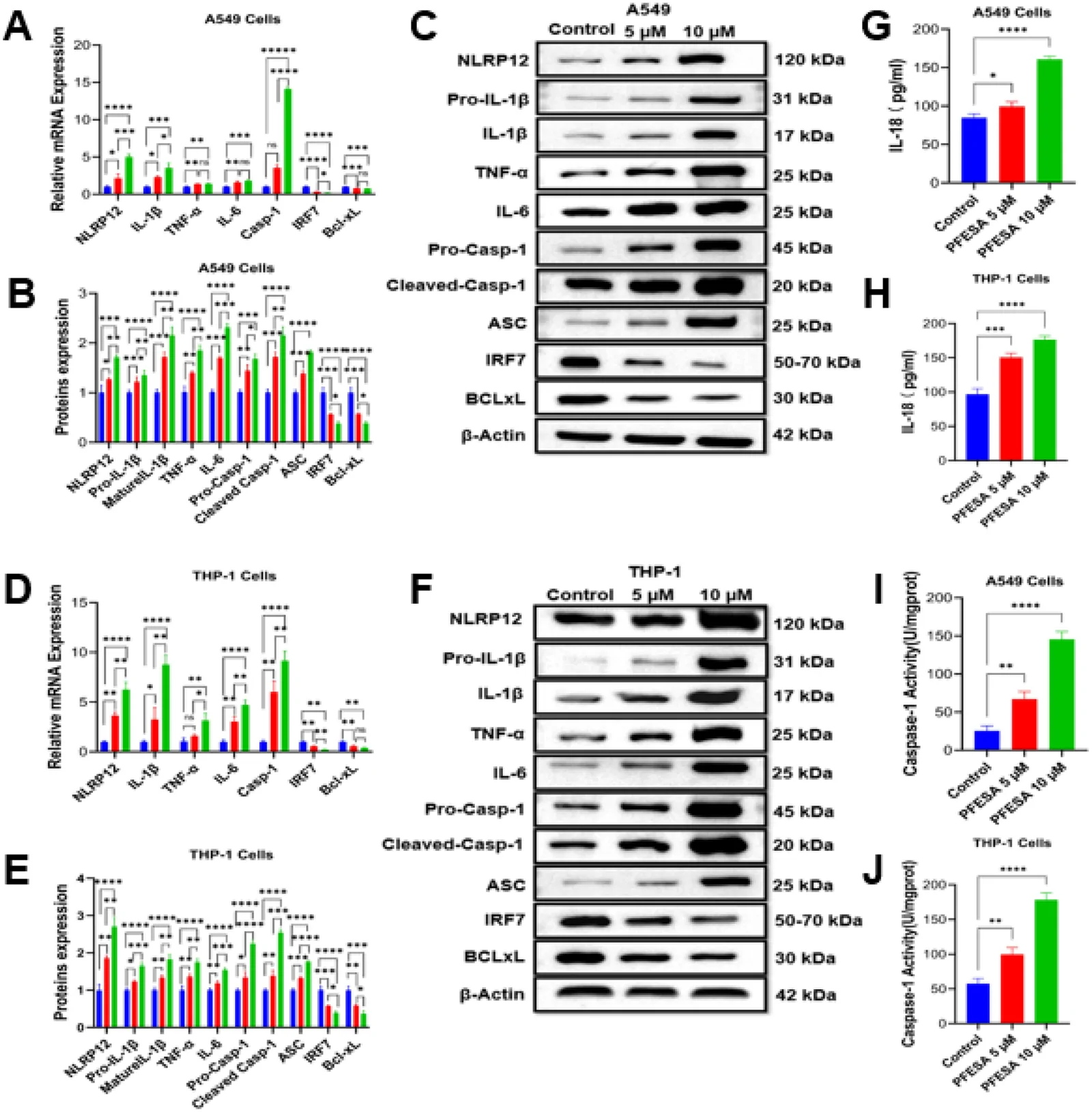
6:2 Cl-PFESA induces inflammasome-associated inflammatory responses in A549 and THP-1 cells. **(A, D)** Relative mRNA expression of *NLRP12, IL-1β, TNF-α, IL-6, Caspase-1, IRF7*, and *Bcl2l1* in A549 and THP-1 cells, respectively. **(B, E)** Quantification of NLRP12, pro-IL-1β, mature IL-1β, TNF-α, IL-6, pro-caspase-1, cleaved caspase-1, ASC, IRF7, and BCL-xL protein levels in the respective cell types. **(C, F)** Representative Western blots of the corresponding proteins; β-actin served as the loading control. **(G, H)** IL-18 secretion measured by ELISA in A549 and THP-1 cells, respectively. **(I, J)** Caspase-1 activity in A549 and THP-1 cells, respectively. Data are presented as mean ± SD (n = 3 independent biological replicates) and analyzed by one-way ANOVA. ns, not significant; *p < 0.05; **p < 0.01; ***p < 0.001; ****p < 0.0001.

Consistent with these transcriptional changes, Western blot analysis revealed increased NLRP12, pro-IL-1β, mature IL-1β, TNF-α, IL-6, pro-caspase-1, cleaved caspase-1, and ASC protein levels in both A549 and THP-1 cells following 6:2 Cl-PFESA exposure. Conversely, IRF7 and BCL-xL protein levels were reduced (**Figures 5B, C, E, F**). The concomitant increases in NLRP12, ASC and cleaved caspase-1, together with enhanced mature IL-1β, support activation of inflammasome-associated signaling in both cell types.

Functional measurements further showed significantly increased IL-18 secretion and caspase-1 activity in both A549 and THP-1 cells, with greater increases at 10 μM than at 5 μM (**Figures 5G–J**). Collectively, these findings demonstrate that 6:2 Cl-PFESA promotes concentration-dependent NLRP12-associated inflammasome activation in both A549 and THP-1 cells, characterized by increased NLRP12 and ASC expression, caspase-1 activation, and enhanced production of the inflammasome-associated cytokines IL-1β and IL-18.

#### 3.5.2 *NLRP12* silencing attenuates 6:2 Cl-PFESA-induced inflammation

Following the identification of *NLRP12* as a potential upstream regulator of 6:2 Cl-PFESA-induced inflammatory signaling, we further investigated its functional involvement. Two independent siRNAs targeting *NLRP12* (siNLRP12–1 and siNLRP12-2) were assessed for knockdown efficiency in both cell models. Both siRNAs significantly reduced *NLRP12* mRNA and protein expression compared with the siNC control, with siNLRP12–1 consistently showing the stronger silencing effect. Overall, siNLRP12–1 reduced *NLRP12* expression by approximately 60-70% at both the mRNA and protein levels, whereas siNLRP12–2 produced a more moderate reduction (**Supplementary Figures S3A–F**). Based on its superior and consistent knockdown efficiency across both cell models, siNLRP12–1 was selected for subsequent functional analyses.

To rigorously dissect the respective contributions of 6:2 Cl-PFESA exposure and NLRP12 depletion, we performed a complete 2 × 2 factorial experiment with four groups: siNC + DMSO, siNC + 10 μM 6:2 Cl-PFESA, siNLRP12 + DMSO, and siNLRP12 + 10 μM 6:2 Cl-PFESA. As expected, 6:2 Cl-PFESA exposure alone markedly increased the mRNA expression of *NLRP12*, *IL1B*, *CASP1*, and *IL18* in both A549 and THP-1 cells (**Figures 6A, D**). Consistently, NLRP12, pro-IL-1β, mature IL-1β, pro-caspase-1, cleaved caspase-1, and ASC protein levels were also elevated, indicating activation of inflammasome-associated inflammatory signaling (**Figures 6B, C, E, F**). Importantly, *NLRP12* silencing substantially attenuated the PFESA-induced inflammatory response, as evidenced by reduced *NLRP12*, *IL1B*, *CASP1*, and *IL18* mRNA expression in siNLRP12 + PFESA cells compared with siNC + PFESA cells (**Figures 6A, D**). This attenuation was also observed at the protein level, with decreased NLRP12, pro-IL-1β, mature IL-1β, pro-caspase-1, cleaved caspase-1, and ASC abundance following NLRP12 depletion (**Figures 6B, C, E, F**). The attenuation was particularly pronounced at the protein level, where most inflammasome-related proteins approached or fell below the levels observed in siNC controls.

**Figure 6:**
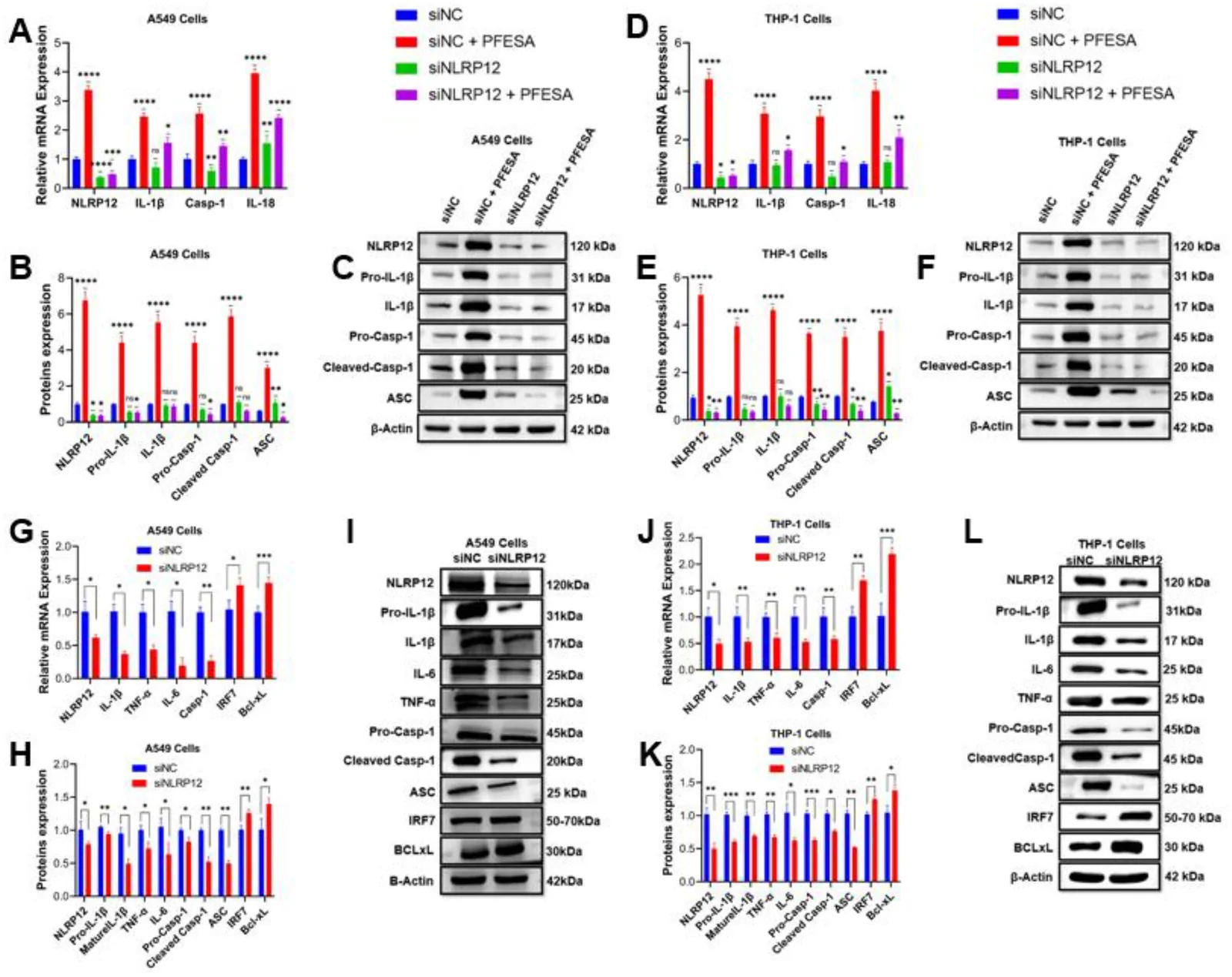
*NLRP12* knockdown attenuates 6:2 Cl-PFESA-induced inflammatory signaling in A549 and THP-1 cells. **(A, D)** qRT-PCR analysis of *NLRP12, IL1B, CASP1*, and *IL18* in A549 and THP-1 cells under the 2 × 2 design: siNC, siNC + 10 μM 6:2 Cl-PFESA, siNLRP12, and siNLRP12 + 6:2 Cl-PFESA. **(B, C, E, F)** Densitometric quantification and representative Western blots of NLRP12, pro-IL-1β, mature IL-1β, pro-caspase-1, cleaved caspase-1, and ASC. **(G, J)** qRT-PCR analysis of *NLRP12, IL1B, TNF, IL6, CASP1, IRF7*, and *BCL2L1* in siNC- and siNLRP12-transfected A549 and THP-1 cells. **(H, I, K, L)** Quantification and representative Western blots of the corresponding proteins. β-Actin was used as the loading control. Data are mean ± SD (n = 3). Panels **(A–F)** were analyzed by two-way ANOVA and panels **(G–L)** by an unpaired two-tailed t-test. ns, not significant; *p < 0.05; **p < 0.01; ***p < 0.001; ****p < 0.0001.

NLRP12 depletion alone also modulated basal inflammatory signaling, reducing the expression of *IL1B*, *TNF*, *IL6*, and *CASP1* in both cell types (**Figures 6G, J**). Correspondingly, the protein levels of IL-1β, TNF-α, IL-6, caspase-1, and ASC were decreased following NLRP12 knockdown (**Figures 6H, I, K, L**). In contrast, *IRF7* and *BCL2L1* expression, together with IRF7 and BCL-xL protein levels, was increased after NLRP12 silencing (**Figures 6G–L**).

Collectively, these findings indicate that NLRP12 is an important positive regulator of 6:2 Cl-PFESA-induced inflammatory signaling in both A549 and THP-1 cells, primarily through modulation of the IL-1β/caspase-1 inflammasome axis, with concomitant regulation of *IRF7* and *BCL2L1*.

### 3.6 6:2 Cl-PFESA promotes lung microbiota dysbiosis

#### 3.6.1 Alterations in microbial diversity and community structure

To characterize the composition and diversity of the lung microbiota following exposure to 6:2 Cl-PFESA 10 mg/kg/body weight, 16S rRNA gene sequencing was conducted on lung tissue samples. A total of 1406 ASVs were identified across all groups, representing the overall microbial diversity captured. The overlapping and exclusive ASVs among the four experimental groups are shown in a Venn diagram (**Figure 7A**). Of these, 178 ASVs were shared across all groups, regardless of sex or treatment. The maximum number was found in the exposed females (n=269). The control male, control female, and exposed males had 237, 65, and 52 unique ASVs, respectively. Additionally, 23 ASVs were present in both male and female groups, suggesting that these microbial taxa are affected by 6:2 Cl-PFESA regardless of sex. Overlap between the control and exposed groups of the same sex reaffirmed the role of biological sex and chemical exposure in the colonization of microbial communities. Interestingly, 6:2 Cl-PFESA exposure reduced ASV richness in males while enhancing it in females, demonstrating sex-dependent effects on microbial diversity (**Figure 7A**). This underscores the need to consider sex as one of the most significant biological factors in microbiome research. Furthermore, the Shannon and Simpson indices were reduced significantly ((*p* < 0.001)) in control and exposed males, with considerable differences between exposed males and females. Conversely, control and treated females did not differ significantly, showing a large reduction in microbial diversity in males (**Figures 7B, C**). Similarly, Chao1, ACE, and observed ASVs (**Figures 7D–F**) exhibited significant ((*p* < 0.05)) reductions in both control and treatment groups, with males showing a substantial reduction. These findings indicate greater vulnerability to microbiota disruption in male mice following exposure to 6:2 CL-PFESA, as reflected by decreased species richness and evenness. The wide variance in diversity metrics within the PFESA-Male group (**Figure 7**) reflects inter-individual heterogeneity in microbial susceptibility, potentially due to differential toxicokinetics, baseline composition, or stochastic ecological processes. In addition, both principal coordinate analysis (PCoA) (explaining 30.29% and 13.98% of the variation, respectively) and non-metric multidimensional scaling (NMDS) (Stress=0.12) (**Figures 7G, H**), respectively, detected significant beta diversity differences among groups (PERMANOVA: R2 = 0.397; *P* = 0.001), indicating profound compositional shifts in the lung microbiota following exposure.

**Figure 7:**
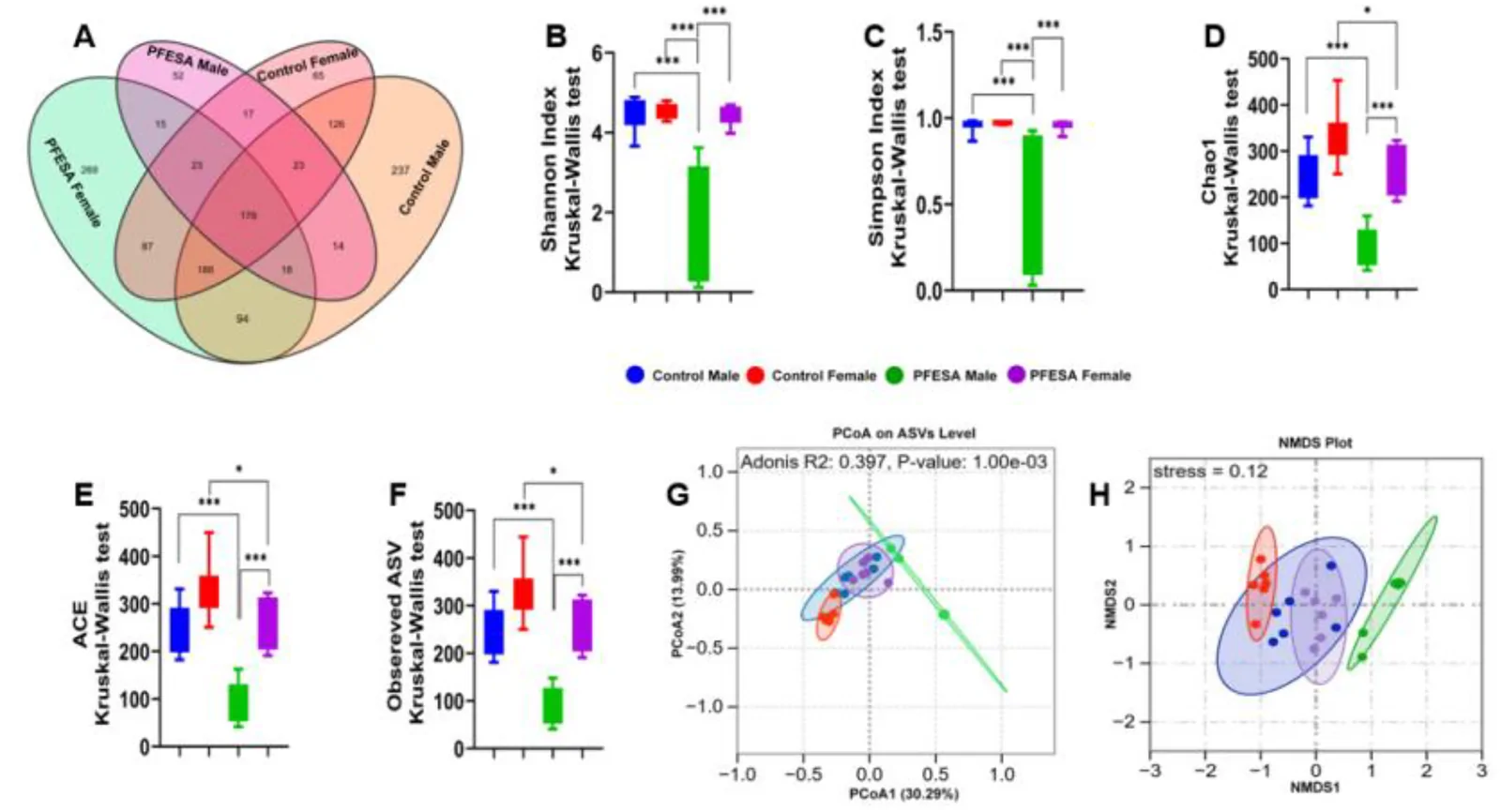
Changes in lung microbiota diversity following exposure to 6:2 Cl-PFESA. (n = 6 independent biological replicates per group). **(A)** ASV-based Venn diagram showing core and unique ASVs among controls and exposed mice. Species diversity is represented using Box plots, which capture the maximum, minimum, dispersion, outliers, and median, with **(B–F)** Shannon, Simpson, Chao1, ACE, and Observed ASV indices, respectively. **(G)** PCoA analysis of lung microbiota based on Bray-Curtis distance. Samples with closer distances indicate more similar species composition, while samples with significant community differences are distinctly separated **(H)**. NMDS analysis of lung microbiota using the Bray-Curtis distance reveals differences in microbial community structure between groups. Dots represent samples; the degree of variation is indicated by the distance between the dots. Samples in the same group are represented with the same color. The significance of intergroup differences in species diversity was assessed using the Kruskal-Wallis test; data are presented as individual sample values with group means. ns, non-significant; (*p < 0.05; **p < 0.01; ***p < 0.001).

#### 3.6.2 Effect of 6:2 Cl-PFESA on lung microbiota composition

To characterize the compositional alterations in the lung microbiota following exposure to 6:2 Cl-PFESA 10 mg/kg body weight exposure, we analyzed its relative composition at the phylum, genus, and species levels (**Supplementary Table S7**). The top 10 phyla, genera, and species are shown in **Supplementary Figures S4A–C**. At the phylum level, the relative abundance of *Proteobacteria*, which are commonly associated with inflammation, was significantly (*p* < 0.01) increased in both exposed groups, with a more pronounced elevation in males. Conversely, the abundance of *Firmicutes* and *Bacteroidetes* was significantly (*p* < 0.01) reduced, with a greater decline detected in males (**Figures 8A–C**). At the Genus level, the opportunistic pathogens *Stenotrophomonas* and *Chryseobacterium* were enriched in exposed mice, with *Stenotrophomonas* showing a more pronounced increase in males. In contrast, the abundance of *Blautia*, generally exhibiting anti-inflammatory effects, was considerably reduced in the exposed groups, with a marked reduction in males (**Figures 8D–F**). At the species level, the abundance of *Stenotrophomonas maltophilia* (*S. maltophilia*) and *Chryseobacterium indologenes* (*C. indologenes*) was both increased (*p* < 0.05) in exposed mice, though only *S. maltophilia* exhibited a significant and male-biased elevation in males (**Figures 8G, H**).

**Figure 8:**
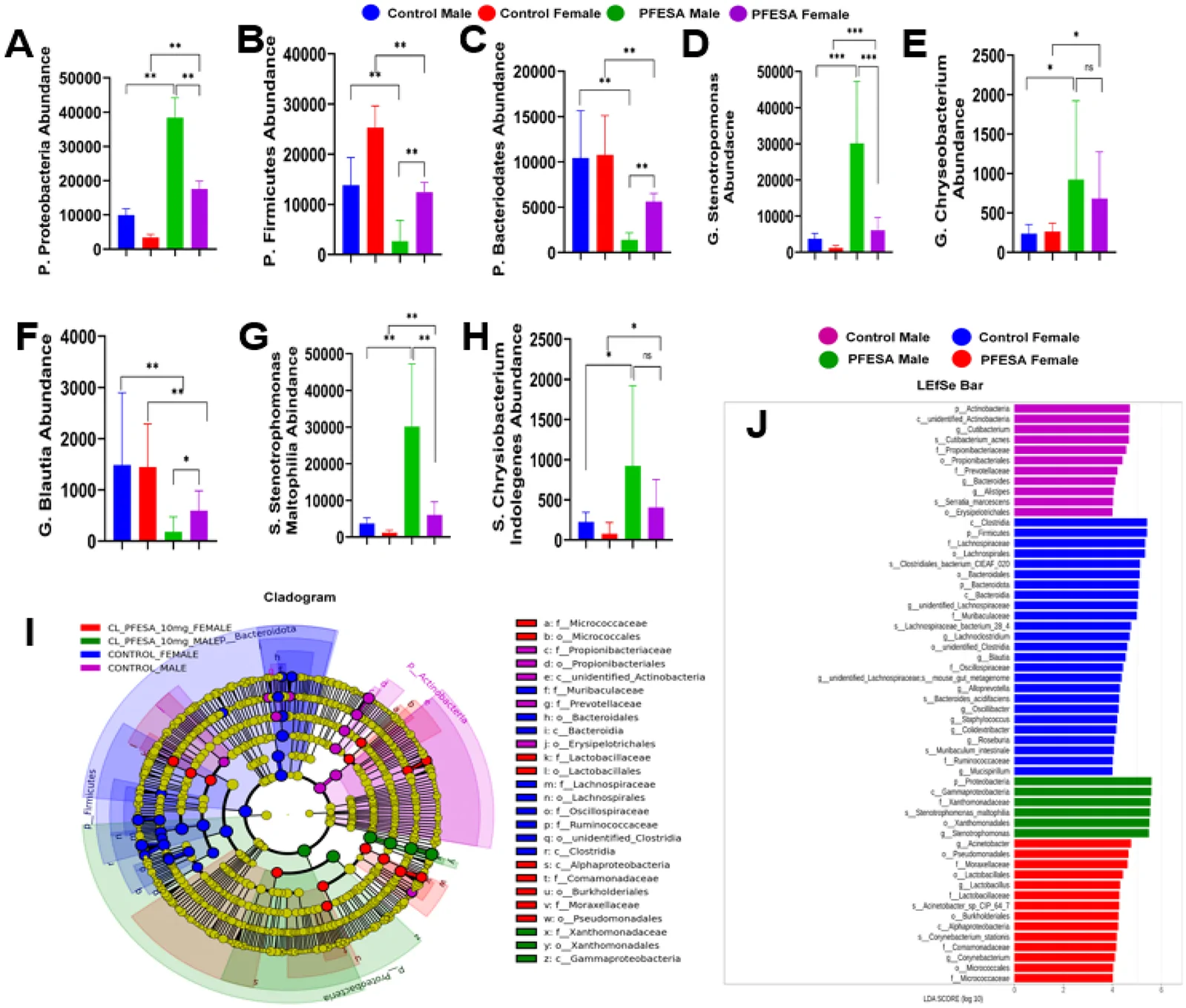
6:2 Cl-PFESA significantly altered lung microbiota composition. (n = 6 independent biological replicates per group). **(A–C)** Phylum, **(D–F)** genus, and **(G, H)** species levels. Metastats was used to analyze and compare the abundance of microbial taxa. Data are represented as individual sample values with group means. Statistical significance between groups is indicated as *p < 0.05, **p < 0.01, and ***p < 0.001. The cladogram and LDA scores illustrate differences in microbial taxa among mouse groups. **(I)** The cladogram displays the distribution of microbial taxa, ranging from phylum to genus/species. Every individual circle at each level represents a specific taxon, with the size of the circle reflecting its relative abundance. Yellow color indicates taxa with no significant differences, while red nodes highlight taxa that are key in the red groups. Green nodes indicate taxa important in the green groups. The legends on the right show the species name. **(J)** Microbial taxa with significant abundance differences between groups are indicated by LDA score (log10 scale, LDA score > 4.0, *P* < 0.05).

LEfSe analysis was conducted to evaluate the contribution of each bacterial species relative abundance to the differences noted in the lung microbiota of the exposed groups (**Figures 8I, J**). A total of 56 differential taxa (*p* < 0.05, LDA>4.0) were identified across all groups. Specifically, 11 taxa were enriched in control males, 25 in control females, 6 in 6:2 Cl-PFESA-exposed males, and 14 in 6:2 Cl-PFESA-exposed females. Notably, the highest number of differential taxa was observed in control females, while the lowest was in 6:2 Cl-PFESA-exposed males, suggesting that 6:2 Cl-PFESA exposure substantially reduced the number of taxa that distinguish the microbial community, particularly in male mice. Furthermore, KEGG analysis revealed that the ABC transporters, DNA repair and recombination proteins, and ribosome pathways were downregulated in both sexes. In contrast, secretion systems-related pathways were upregulated in both groups. In particular, males displayed elevated enzyme activity and two-component systems and reduced tRNA biogenesis, whereas females exhibited reduced peptidase and inhibitory responses (**Supplementary Figure S5**). These results reveal sex-specific differences in microbial response to 6:2 Cl-PFESA.

### 3.7 *S. maltophilia* challenge enhances *NLRP12*-associated inflammatory signaling in PFESA-exposed A549 cells

To investigate the functional relationship between *S. maltophilia* and *NLRP12*-associated inflammatory signaling, A549 cells were exposed to *S. maltophilia* alone, PFESA alone, or PFESA pre-exposure followed by *S. maltophilia* challenge. Both *S. maltophilia* and PFESA significantly increased the mRNA expression of *NLRP12, IL-1β, IL-18*, and *Caspase-1* compared with the control (**Figures 9A–D**). Notably, PFESA pre-exposure followed by *S. maltophilia* challenge produced the highest expression levels across all four inflammatory markers. Compared with PFESA alone, the PFESA + SMA group showed significantly higher expression of *NLRP12* (p < 0.01), *IL-1β* (p < 0.01), *IL-18* (p < 0.01), and *Caspase-1* (*p* < 0.001). Similarly, compared with *S. maltophilia* alone, combined PFESA + SMA exposure significantly increased *NLRP12* (*p* < 0.05), *IL-18* (*p* < 0.05), and *Caspase-1* (*p* < 0.001), whereas the increase in *IL-1β* was not statistically significant. No significant differences were observed between the SMA-alone and PFESA-alone groups for *NLRP12, IL-18*, or *Caspase-1*.

**Figure 9:**
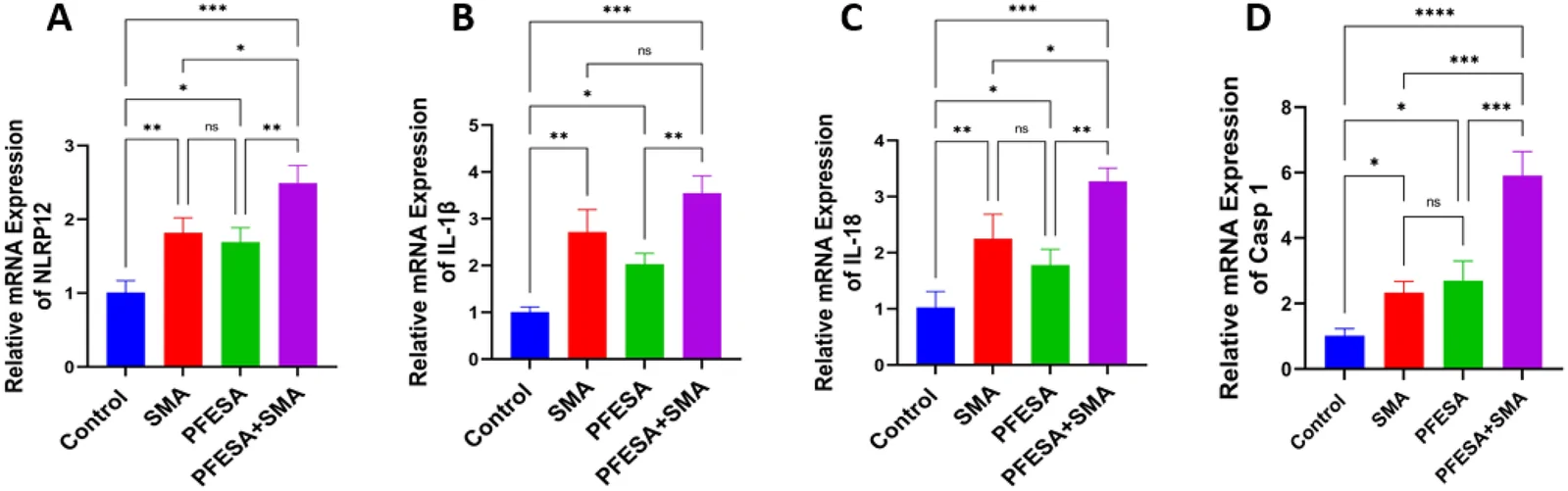
*S. maltophilia* enhances PFESA-induced inflammatory signaling in A549 cells. Relative mRNA expression of **(A)** *NLRP12*, **(B)** *IL-1β*, **(C)** *IL-18*, and **(D)** *Caspase-1.* Data are presented as mean ± SD (n = 3 independent biological replicates per group). Data were analyzed by two-way ANOVA: ns, non-significant; *p < 0.05; **p < 0.01; ***p < 0.001, ****p < 0.001).

Collectively, these results demonstrate that *S. maltophilia* induces *NLRP12-* and inflammasome-associated transcriptional responses in A549 cells and that prior PFESA exposure further enhances the response to subsequent bacterial challenge.

### 3.8 Correlation between gene expression and microbial species abundance

Based on the RNA-seq findings (Section 3.3) and qRT-PCR validation (Section 3.4), we selected a panel of differentially expressed inflammatory and immune-regulatory genes for correlation analysis with microbial abundance. Spearman’s rank correlation was performed to assess pairwise associations between gene expression levels and bacterial species abundance in male and female mice separately (**Figures 10A, B**).

**Figure 10:**
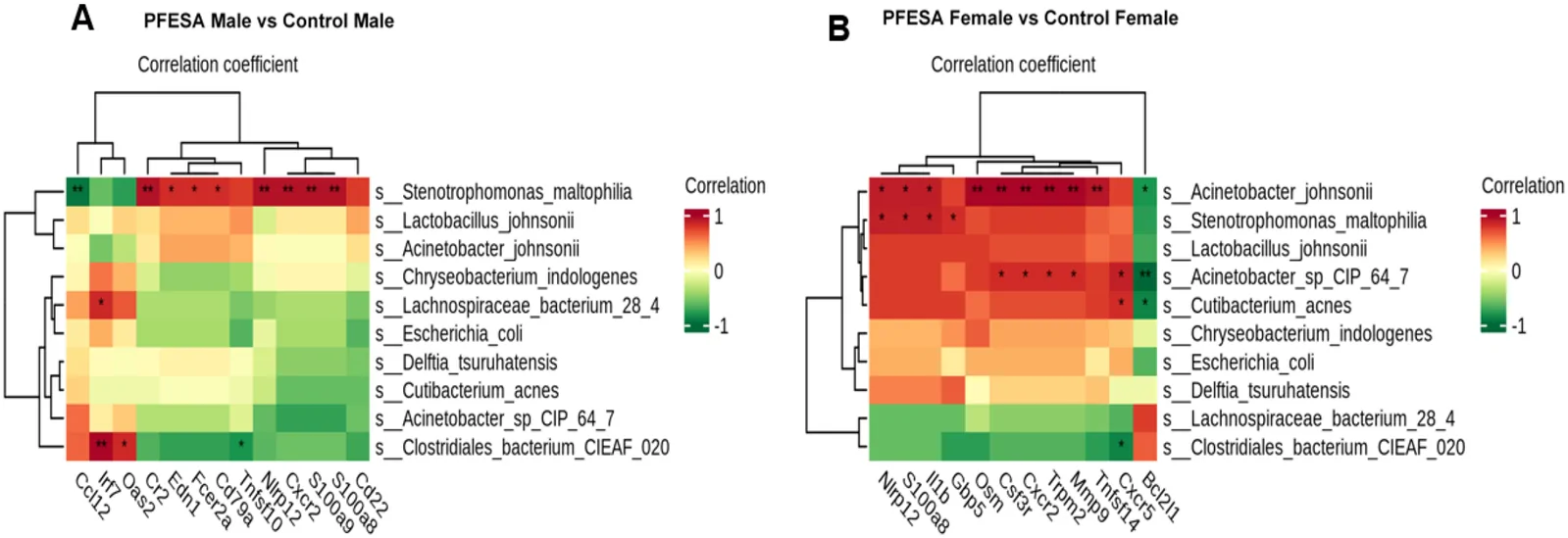
Spearman’s correlation analysis of the top 10 bacterial species and the inflammatory genes of the top 25 KEGG pathways. **(A)** PFESA male vs control male **(B)** PFESA female vs control female. Statistical significance is denoted as: *p < 0.05, and **p < 0.01.

In male mice (**Figure 10A**), strong positive correlations (*p* < 0.01, shown in red) were identified between pro-inflammatory genes (*NLRP12, S100a8, S100a9*, and *Cd22*) and the opportunistic pathogen *S. maltophilia*. Conversely, genes associated with antiviral defense and immune regulation (*Irf7, Oas2, Ccl12, FcϵRIα*, and *Tnfsf10*) exhibited negative correlations with both pathogenic and commensal bacteria. For instance, *IRF7* was negatively correlated with *S. maltophilia*, suggesting that suppression of antiviral signaling may be associated with this pathogen’s proliferation. Furthermore, *Tnfsf10, FcϵRIα*, and *NLRP12* were negatively correlated with beneficial bacteria such as *Acinetobacter* sp. *CIP_64_7, Clostridiales bacterium CIEAF_020*, and *Lachnospiraceae bacterium 284*, suggesting that the loss of beneficial microbes may accompany the upregulation of pro-inflammatory responses.

The same trend was observed in female mice (**Figure 10B**). There were positive associations of inflammatory genes (*NLRP12, IL-1β, Gbp5*, and *Trpm2*) with multiple bacterial taxa, including opportunistic bacteria such as *Acinetobacter johnsonii, S. maltophilia*, and *Cutibacterium acnes*, as well as the commensal *Lactobacillus johnsonii*. Remarkably, these genes displayed negative correlations with potentially protective species such as *Clostridiales* bacterium CIEAF_020 and *Lachnospiraceae* bacterium 284. Furthermore, *Bcl2l1* was negatively correlated with *Acinetobacter johnsonii* and *S. Maltophilia*, suggesting that higher abundance of these potentially pathogenic species may be associated with reduced anti-apoptotic signaling in the female lung.

Collectively, these correlation analyses revealed significant associations between specific microbial taxa-particularly the male-biased pathogen *S. maltophilia*-and key inflammatory and immune-regulatory genes, supporting the hypothesis that sex-dependent microbial dysbiosis may contribute to the differential lung inflammatory responses observed in male and female mice following 6:2 Cl-PFESA exposure.

## 4 Discussion

The safety case for any PFOS replacement rests on the assumption that the organs and pathways it damages are already known. 6:2 Cl-PFESA has been confirmed to be associated with toxicity in multiple organs (7). However, the specific mechanism by which it causes lung inflammation remains unclear. Our findings challenge that assumption for 6:2 Cl-PFESA. By combining *in vivo* phenotyping, lung transcriptomics, microbial profiling, and loss-of-function experiments, we identify the lung as a target organ of 6:2 Cl-PFESA, define *NLRP12* as the convergent innate immune node through which chemical stress is transduced into inflammation, and show that the severity of injury is sex-dependent and tracks the magnitude of pulmonary microbial dysbiosis rather than the breadth of the transcriptional response.

Toxicological attention to 6:2 Cl-PFESA has centered on the liver and kidney (27, 28). Our data place the lung alongside them. Twenty-eight days of oral exposure produced concurrent body-weight loss, alveolar collapse, septal thickening, emphysematous change, and dense inflammatory infiltration, together with a systemic pro-inflammatory shift, elevated serum IL-1β, IL-6, and TNF-α with reciprocal suppression of IL-4 and IL-10. Each of these endpoints was more severe in males, and the sex difference was both temporal and quantitative: weight loss emerged by day 7 in males but by day 14 in females. Because the compound was delivered by gavage rather than by inhalation, the pulmonary phenotype cannot be attributed to direct airway deposition; it indicates that systemically distributed 6:2 Cl-PFESA reaches and injures the alveolar compartment, and that the lung is therefore a target of internal exposure in the general and occupationally exposed population, not merely of aerosol contact.

The present data demonstrate that sex-dependent susceptibility to 6:2 Cl-PFESA-induced pulmonary inflammation manifests as divergent response intensity and kinetics. Females exhibited earlier onset and greater magnitude of inflammatory gene upregulation, whereas males displayed a more progressive dysbiotic shift together with pronounced oxidative stress, suggesting that sex modulates the host-microbiome-inflammatory axis at multiple interconnected levels. Among the endpoints assessed, pulmonary LPS accumulation emerged as the most mechanistically informative. In exposed lungs, increased tissue endotoxin levels in the absence of overt infection serve as a hallmark of barrier dysfunction and indicate an increased Gram-negative bacterial burden, providing a critical priming signal for NLR-dependent inflammasome activation. Notably, this accumulation was disproportionately greater in males. This observation suggests that the sex difference may present not two distinct phenotypes, but rather a difference in the intensity of a shared endotoxin-driven stimulus, upon which established sex differences in energy metabolism, xenobiotic disposition, and immune regulation are superimposed (29). Several candidate mechanisms may underpin these observations. First, sex-dependent toxicokinetics may contribute to the differential responses. Li et al. demonstrated sex-related differences in the acute toxicity and *in vivo* distribution of 6:2 Cl-PFESA in rats, indicating that systemic exposure and tissue partitioning differ between males and females. Such sex-dependent toxicokinetic differences could potentially contribute to the distinct inflammatory response patterns observed in our study, although the relationship between tissue distribution and the timing or severity of inflammation remains to be established (16). Second, estrogen signaling through ERβ modulates mucosal immune tolerance and regulatory T cell function; Goodman et al. demonstrated that impaired estrogen signaling underlies regulatory T cell loss-of-function in the chronically inflamed intestine, with ERβ-specific signaling required for TGF-β-dependent Treg differentiation, a pathway that may paradoxically both protect against and delay inflammatory responses depending on exposure context (30). Third, sex hormones shape gut microbial composition through bidirectional crosstalk; females generally exhibiting higher alpha diversity and abundance of immunoregulatory taxa such as *Akkermansia* and *Bifidobacterium* (31), while the gut microbiome and sex steroids engage in bidirectional regulation via the so-called “estrobolome” (32). Importantly, while our data directly support the observed sex differences in inflammatory onset, dysbiosis magnitude, and redox kinetics, the underlying causal mechanisms remain speculative. Our correlational dataset cannot distinguish whether the observed effects arise from direct PFAS-induced epithelial injury, microbiome-mediated immune modulation, or their interaction, nor can it resolve whether hormonal, genetic, or microbiome-driven factors are primary. Nevertheless, our findings establish sex as an integral biological variable shaping 6:2 Cl-PFESA-induced pulmonary pathology, with implications for risk assessment and therapeutic stratification.

Alveolar epithelial oxidative injury is the most primitive and most preserved response of the alveolar epithelium to chronic exposure to fluorinated chemicals. Reactive oxygen species (ROS) are major mediators of oxidative stress, leading to DNA damage and lipid peroxidation (33), and PFOS has been shown to increase ROS to cytotoxic levels (34–36). The same pattern was observed at the organismal and cellular level for 6:2 Cl-PFESA, and the direction of change across individual markers is more informative than their statistical significance alone. In both sexes, the levels of MDA, which is a final product of the lipid peroxidation process, increased and even more so in males, suggesting that oxidative damage had already been accomplished, not just threatened. Concomitantly, CAT and GSH increased, interpreted as a compensatory increase in antioxidant system in the face of persistent oxidant load, while the parallel decrease in superoxide dismutase activity, again most pronounced in males, suggests exhaustion of the capacity of first-line antioxidant dismutase. The net signature is a redox system that is engaged, overwhelmed, and failing, and it fails earlier in males. The capacity of 6:2 Cl-PFESA to perturb oxidative and antioxidative enzymes across phylogenetically distant organisms, including zebrafish (37) and chlorella (38), indicates that this is an intrinsic property of the molecule rather than a species-specific artefact.

Mitogen-activated protein kinases (MAPKs), including *p38*, *ERK*, and *JNK*, are major components of the cellular response to chemical and oxidative stress (35). In A549 and THP-1 cells, 6:2 Cl-PFESA elicited a dose-dependent rise in intracellular ROS accompanied by downregulation of *p38* and upregulation of *ERK* and *JNK*, a directional pattern consistent with the reported capacity of PFAS, including 6:2 Cl-PFESA, to bind MAPK proteins and form stable interactions. Loss of *p38* signaling removes a principal brake on inflammatory gene transcription, while sustained *ERK* and *JNK* activity drives stress-adaptive and pro-inflammatory programs. The downstream response, coordinated induction of *Nrf2*, *HIF-1*, and *VEGFA*, indicates that the antioxidant and hypoxia-adaptive arms were engaged simultaneously (39). *Nrf2* induction is a classical protective response, but its coincidence with rising MDA and falling SOD *in vivo* demonstrates that this defense was mounted and outpaced. Redox failure therefore represents the proximal epithelial insult, not the terminal one, and its principal consequence is a barrier that can no longer exclude microbial products.

The lung transcriptome revealed a striking dissociation between the breadth of the response and its consequences. Female lungs mounted a substantially larger transcriptional response (209 differentially expressed genes) than male lungs (88), with only 21 genes (7.6%) shared between the sexes, yet the males sustained the greater injury. The number of differentially expressed genes is thus not a proxy for toxicity. We interpret the expansive female response as reflecting greater transcriptional plasticity and a broader compensatory mobilization, whereas the narrower male program is dominated by inflammatory effectors on a background of higher endotoxin load and deeper redox failure, a smaller but more permissive response. This distinction has direct implications for study design: sex-pooled transcriptomic screens will systematically obscure precisely the axis that determines susceptibility.

Against this divergent background, one pathway was shared. KEGG enrichment identified the NOD-like receptor signaling pathway in both sexes, and within it *NLRP12* was consistently upregulated in males and females alike, a sex-independent node acting on a sex-dependent substrate, and therefore the natural candidate for mechanistic dissection. *NLRP12* has a well-documented role in innate immune responses to infection: it supports caspase-1 activation and the release of IL-1β and IL-18 in *Yersinia pestis* (40) and *Plasmodium chabaudi* (41), models, promotes neutrophil chemotaxis by stabilizing *CXCL1* mRNA during influenza infection (42), participates in inflammasome assembly during early HSV-1 infection (43), and interacts with the SARS-CoV-2 M protein to amplify inflammation (44). Its role, however, is genuinely context-dependent, since *NLRP12* also acts as a negative regulator of canonical and non-canonical NF-κB signaling in several myeloid settings. This duality makes correlative upregulation insufficient as evidence, and it is the reason we tested the gene directly. siRNA-mediated knockdown of *NLRP12* in A549 and THP-1 cells markedly attenuated the 6:2 Cl-PFESA-induced induction of IL-1β, IL-6, TNF-α, and caspase-1 at both transcript and protein level, and simultaneously restored *IRF7* and *BCL2L1* expression. Importantly, beyond transcriptional regulation, our additional functional analyses demonstrated that 6:2 Cl-PFESA exposure induced NLRP12 inflammasome-associated activation in A549 and THP-1 cells. PFESA treatment enhanced NLRP12 protein expression, promoted the cleavage of pro-caspase-1 into active caspase-1, and increased the processing of pro-IL-1β into mature IL-1β. Furthermore, elevated IL-18 secretion and increased caspase-1 enzymatic activity confirmed functional activation of the inflammasome pathway. These findings demonstrate that PFESA-induced NLRP12 upregulation is accompanied by downstream inflammasome activation rather than representing only a compensatory transcriptional response. *NLRP12* therefore functions as a positive, non-redundant mediator of 6:2 Cl-PFESA-induced inflammation in the alveolar epithelium, and it sits upstream of, rather than parallel to, the sex-restricted effectors described below, further indicating that the receptor is a general integrator of inhaled and systemic chemical stress in the lung, of which 6:2 Cl-PFESA is now a defined activator.

As a key transcription factor, *IRF7* regulates the balance between pro- and anti-inflammatory responses through multiple routes, including autophagy and NF-κB signaling, and can restrain autophagy via the RAPTOR/mTORC1 axis, thereby exacerbating inflammatory tissue damage (45). In our study, *IRF7* was downregulated exclusively in exposed males. The predicted consequence is the release of NF-κB from *IRF7*-dependent restraint and the unchecked secretion of TNF-α and IL-6 (46), precisely matches the male-biased serum cytokine profile, and the negative correlation between *IRF7* and *Stenotrophomonas maltophilia* abundance ties this loss of regulatory tone directly to the dysbiotic community described below. In females, by contrast, the anti-apoptotic gene *BCL2L1* (encoding Bcl-xL), which constrains cell death and inflammation by inhibiting caspase-1 (47), was selectively downregulated, a change expected to de-repress caspase-1-dependent inflammatory signaling. The two sexes therefore route a common upstream signal (*NLRP12*) into distinct effector arms: loss of transcriptional restraint on NF-κB in males, loss of post-translational restraint on caspase-1 in females, and the restoration of both genes upon *NLRP12* knockdown places them downstream of the same node. That both arms are inflammatory, yet only males develop severe injury, indicates that the effector identity alone does not determine outcome; what differs is the intensity of the upstream stimulus.

The sex-specific restructuring of the lung microbiome observed in this study points to a microbially derived source of differential inflammatory stimulus. PFAS are known to alter host-associated microbial communities (48); however, the lung microbiome has not been studied following exposure to any PFAS alternative. In exposed animals, Shannon and Simpson indices decreased and NMDS and PCoA ordination separated exposed from control communities, reflecting a real restructuring, not a random drift. The observed sex-specific variation reduced ASV richness in males versus enhanced richness in females likely reflects the interplay of hormonal, metabolic, and immune factors. Sex-dependent differences in PFAS toxicokinetics are well-established: female rodents exhibit more rapid renal clearance due to sex-specific organic anion transporter expression, resulting in shorter elimination half-lives compared to males (49). Additionally, estrogen modulates gut microbial composition by influencing the gut environment and promoting beneficial genera such as *Bifidobacterium* and *Akkermansia* (50), whereas androgens exert immunosuppressive effects and alter colonization resistance (32). A systematic review further established that females generally exhibit higher alpha diversity and greater abundance of immunoregulatory taxa (50). These factors, together with sex-specific differences in immune function and metabolic regulation, may collectively underpin the divergent microbial responses observed following 6:2 Cl-PFESA exposure, underscoring the need to consider sex as a fundamental biological variable in microbiome research. At the phylum level, *Proteobacteria*, which have been linked with inflammatory conditions previously (51), increased in both sexes and especially in males, while *Firmicutes* and *Bacteroidetes*, which have been linked with community stability and anti-inflammatory tone (52, 53) were depleted. At the genus level, opportunistic pathogens *Stenotrophomonas* and *Chryseobacterium* were enriched (54), whereas *Blautia*, which has anti-inflammatory signaling and mucosal integrity activity (55), was depleted, especially in males. The species-level feature most discriminating between the two groups was the male-biased expansion of *S. maltophilia*, an opportunistic pathogen of worldwide importance and a known cause of pneumonia (56). In parallel, but without the male bias, the growth of *Chryseobacterium indologenes* increased, which further supported the conclusion that the sex-specific signal is not due to opportunistic overgrowth as a whole but is specific to *S. maltophilia.* The targeted *S. maltophilia* challenge experiment provided functional support for the microbiome-inflammation relationship identified in the sequencing analysis. *S. maltophilia* alone increased the expression of *NLRP12, IL-1β, IL-18*, and *Caspase-1* in A549 cells, while PFESA pre-exposure followed by bacterial challenge produced the strongest transcriptional response. These findings suggest that PFESA exposure may sensitize pulmonary epithelial cells to subsequent *S. maltophilia* stimulation, thereby amplifying NLRP12-associated inflammatory signaling. This result strengthens the biological plausibility of enriched *S. maltophilia* and aggravated pulmonary inflammation observed *in vivo*.

These microbial and host datasets converge. *S. maltophilia* abundance was correlated positively with *NLRP12, S100a8, S100a9* and *Cd22*, and negatively with *IRF7*, and as a Gram-negative bacterium, it induces inflammatory signaling via LPS and TLR4 (57). This is the same LPS that accumulated in exposed lungs, and it is the canonical priming signal for the NLR pathway that our transcriptomic and knockdown data independently implicate. We therefore propose a multi-faceted, self-reinforcing model: 6:2 Cl-PFESA promotes epithelial redox stress and barrier impairment, which allows for the expansion of Gram-negative bacteria and loss of protective commensals, which primes *NLRP12*-dependent inflammasome signaling; this is followed by the release of *IL-1β* via the inflammasome and further cytokine amplification, which causes additional epithelial and barrier damage and loss, closing a feed-forward loop. Within this framework, the sex difference has a coherent explanation. Males do not possess a distinct injury mechanism; they experience a more intense version of the same one, because their microbial community shifts further toward a Gram-negative, LPS-producing configuration and their redox defenses fail earlier. Crucially, this accounts for the apparent paradox of the transcriptome, since injury severity correlates with the magnitude of dysbiosis and endotoxin exposure rather than with the number of genes mobilized.

Oxidative stress, inflammatory signaling, and microbial dysbiosis may represent interconnected responses to chemically diverse environmental pollutants. Chen et al. recently demonstrated that early-life and perinatal Hypochlorous Acid (HOCl) exposure aggravated airway inflammation and hyperresponsiveness in asthmatic mice, accompanied by reduced gut microbial diversity, altered bacterial composition, and disruption of arachidonic acid metabolism. Moreover, taurine-mediated neutralization of HOCl attenuated these effects, supporting the contribution of oxidative injury to the aggravated airway phenotype (58). Similarly, the present study showed that 6:2 Cl-PFESA-induced pulmonary inflammation was associated with oxidative stress, MAPK activation, NLRP12-related inflammatory signaling, increased pulmonary LPS, and sex-specific lung microbiota dysbiosis. Nevertheless, important mechanistic differences exist between the two exposure models. HOCl is a highly reactive oxidant examined in the context of pre-existing allergic airway inflammation and was primarily associated with a gut microbiota-arachidonic acid metabolic axis. In contrast, 6:2 Cl-PFESA is a persistent fluorinated contaminant whose pulmonary effects in the present study were associated with ROS/MAPK-NLRP12 signaling and alterations in the lung microbiota. Consistent with this interpretation, pulmonary microbiota dysbiosis has also been reported to modulate oxidative stress and inflammatory responses following PM_2.5_ exposure (59). Collectively, these findings suggest that oxidative stress, inflammation, and microbial disruption may constitute shared systems-level responses to environmental pollutants, whereas the molecular initiating events, affected microbial compartments, and downstream pathways remain pollutant- and disease-context dependent.

Several limitations should be acknowledged. First, the main findings were derived from a single dose of 10 mg/kg body weight, selected on the basis of our pilot study, which precluded evaluation of dose-response relationships and exposure thresholds. Although this dose is consistent with those used in previous mouse toxicity studies of 6:2 Cl-PFESA (60), amphibian studies have also reported significant PFAS-induced effects at environmentally relevant concentrations of 1-10 μg/L (39). Future studies should incorporate a graded dose range (e.g., 0.1–10 mg/kg body weight) and employ repeated and chronic exposure designs with larger cohorts and balanced sex distribution to better characterize dose-dependent effects and strengthen environmental risk assessment. Second, although multiple complementary datasets supported sex-dependent susceptibility to 6:2 Cl-PFESA-induced pulmonary inflammation, the underlying mechanisms driving this sex-specific response remain incompletely understood. Differences in sex hormone regulation, toxicokinetics, and immune modulation may contribute to the observed differences; however, these factors were not directly evaluated in the present study. Future investigations integrating hormonal profiling and sex-specific toxicokinetic analyses will be necessary to clarify these mechanisms. Third, although our findings demonstrate caspase-1 activation and downstream inflammatory cytokine maturation, additional analyses of ASC speck formation and gasdermin D cleavage are needed to further confirm canonical inflammasome assembly and pyroptotic signaling in 6:2 Cl-PFESA-induced lung inflammation. Furthermore, while the present study supports ROS-dependent MAPK involvement in 6:2 Cl-PFESA-induced inflammation, the molecular initiating target and primary cellular source of ROS remain unresolved. Future studies combining genetic and pharmacological approaches targeting ROS-generating systems, together with unbiased target identification and direct binding analyses, will be required to define the upstream mechanisms linking 6:2 Cl-PFESA exposure to oxidative stress, MAPK dysregulation, and inflammatory activation. Fourth, the microbial intervention experiments were limited to an *in vitro* A549 cell model and transcriptional endpoints. Although these findings provide functional support for a *S. maltophilia*-*NLRP12* inflammatory link under 6:2 Cl-PFESA exposure, definitive *in vivo* causality remains to be established. Future studies incorporating targeted depletion or recolonization of *S. maltophilia*, together with genetic or pharmacological manipulation of *NLRP12*, will be required to confirm its contribution to PFESA-induced pulmonary inflammation.

## 5 Conclusion

In conclusion, exposure to 6:2 Cl-PFESA leads to sex-dependent lung inflammation and injury in mice, with males exhibiting greater susceptibility. Transcriptomics revealed profound sexual dimorphism, with female lungs showing substantially more differentially expressed genes than males and minimal overlap between sexes. Mechanistically, 6:2 Cl-PFESA-induced NLRP12 inflammasome-associated activation contributes to lung inflammation, as demonstrated by *NLRP12*-dependent regulation of inflammatory mediators together with enhanced caspase-1 cleavage, mature IL-1β processing, IL-18 secretion, and caspase-1 enzymatic activity. 6:2 Cl-PFESA also triggered ROS overproduction and differential MAPK pathway regulation. Notably, exposure induced sex-dependent lung microbiota dysbiosis, characterized by male-biased enrichment of *S. maltophilia* and depletion of *Blautia*, correlating with host inflammatory gene expression. These findings establish *NLRP12* activation and microbial dysbiosis as key mechanisms underlying sex-dependent lung injury, providing a framework for sex-specific risk assessment of PFAS alternatives and identifying *NLRP12* and the lung microbiome as potential therapeutic targets.

## Data Availability

The data presented in this study are depositedin the NCBI Sequence Read Archive (SRA) under BioProject accession number PRJNA1464209. The dataset includes 16S rRNA microbiome sequencing and RNA-sequencing data, with each biological replicate submitted as an individual BioSample within the BioProject.
