# Supplementary material for: 6:2 Cl-PFESA induces sex-dependent lung inflammation through NLRP12 inflammasome activation and pulmonary microbiota dysbiosis
Source: Front Immunol. 2026 Oct 30;17:1939840. doi: 10.3389/fimmu.2026.1939840 (PMC13759367; doi:10.3389/fimmu.2026.1939840)
Supplement: Supplementary file 1 [file DataSheet1.docx]

**6:2 Cl-PFESA Induces Sex-Dependent Lung Inflammation Through NLRP12 Inflammasome Activation and Pulmonary Microbiota Dysbiosis**

Suliman Khan^2,3^, Min Chen^2^, Mubbashar Abbas^2^, Zhen Chen^2^, Zhiwei Liang^2^, Rizwan Ullah^4^, Shuyu Xie^1,5*^, Dongmei Chen^2*^

**Affiliation:**

^1^State Key Laboratory of Agricultural Microbiology Core Facility, Huazhong Agricultural University, Wuhan, Hubei 430070, China

^2^National Reference Laboratory of Veterinary Drug Residues (HZAU), Wuhan, Hubei 430070, China

^3^Department of Veterinary Physiology and Biochemistry, Lasbela University of Agriculture, Water and Marine Science, Uthal, Balochistan, Pakistan.

^4^National Key Laboratory of Agricultural Microbiology, College of Veterinary Medicine, Huazhong Agricultural University, Wuhan, 430070, Hubei, China

^5^Hubei Hongshan laboratory, Wuhan, Hubei 430070, China

**Figure S1** Effects of 6:2 Cl-PFESA exposure on body weight, inflammatory cytokines, and lung histology in mice. (A) Body weight loss in mice following exposure. (B, C, D) Levels of IL-1β, TNF-α, and IL-6 (E). Representative H&E-stained images of lung sections from mice (200x magnification). Data were analyzed by two-way ANOVA, represented as mean ± S.D. ns, non-significant; **p*<0.05; ***p*<0.01; ****p*<0.001; *****p*<0.0001**.**

**Figure S2** (A-B) Cell viability of A549 cells exposed to different concentrations of 6:2 Cl-PFESA for 24 h and 48 h. (C-D) Cell viability of THP-1 cells exposed to different concentrations of 6:2 Cl-PFESA for 24 h and 48 h. Cell viability was determined using the CCK-8 assay. Data are presented as mean ± SD (n = 3). Statistical analysis was performed using Two-way ANOVA. *p* < 0.05, **p* < 0.01, ***p* < 0.001, ****p* < 0.0001**.**

**Figure S3** Validation of *NLRP12* knockdown efficiency in A549 and THP-1 cells.
(A-C) *NLRP12* knockdown efficiency in A549 cells transfected with siNC, siNLRP12-1, or siNLRP12-2. (A) qRT-PCR analysis of *NLRP12* mRNA expression. (B-C) Quantification and representative Western blot analysis of NLRP12 protein expression. (D-F) *NLRP12* knockdown efficiency in THP-1 cells. (D) qRT-PCR analysis of *NLRP12* mRNA expression. (E-F) Quantification and representative Western blot analysis of NLRP12 protein expression. β-Actin was used as the loading control. Data are presented as mean ± SD (n = 3). Statistical analysis was performed using two-way ANOVA. ***p* < 0.001, ****p* < 0.0001**.**

**Figure S4** Relative abundance of lung microbiota at (A) phylum, (B) genus and (C) species levels after exposure to 6:2 Cl-PFESA

**Figure S5** KEGG pathways showing microbial response to 6:2 Cl-PFESA

| **Table S1** Sequences of siRNAs targeting human *NLRP12* | | | |
| --- | --- | --- | --- |
| **siRNA** | **Target gene** | **Strand** | **Sequence (5′→3′)** |
| H91662- siNLRP12-1 | *NLRP12* | sense | UGUUCAUGUUGAGGAAGGC (dT) (dT) |
|  | *NLRP12* | anti-sense | GCCUUCCUCAACAUGAACA (dT) (dT) |
| H91662- siNLRP12-2 | *NLRP12* | sense | UGGAUGUCGUACAAGCAGC (dT) (dT) |
|  | *NLRP12* | anti-sense | GCUGCUUGUACGAGAUCCA (dT) (dT) |

| **Table S2** qRT-PCR primers Sequences | | |
| --- | --- | --- |
| **Gene** | **Sequence (5′-3′)** | **Reference** |
| *NLRP12-F* | GCAGCTCTCATAGCCAATAAG | (Li et al., 2024) |
| *NLRP12-R* | CTGACACTTCCTCAACTGAATC |  |
| *IL-1ß-F* | GGATATGGAGCAACAAGTGG |  |
| *IL-1ß-R* | GAAGTCAGTTATATCCTGGC |  |
| *TNF-α-F* | TGGAGACCATCAAGGAAGAC |  |
| *TNF-α-R* | TCAGCCATCACTTGGATGAG |  |
| *IL-6-F* | ACTCACCTCTTCAGAACGAATTG |  |
| *IL-6-R* | CCATCTTTGGAAGGTTCAGGTTG |  |
| *ß-actin-F* | GCTCCTCCTGAGCGCAAG |  |
| *ß-actin-R* | CATCTGCTGGAAGGTGGACA |  |
| *BCL2L1-F* | TAA GGC GGA TTT GAA TCT C | (Ghaemi et al., 2020) |
| *BCL2L1-R* | ATA ATA GGG ATG GGC TCA AC |  |
| *IRF7-F* | CCCATCTTCGACTTCAGCAC | (Fan et al., 2024) |
| *IRF7-R* | TGTAGTGTGGTGACCCTTGC |  |
| *Casp1-F* | CCCACATCCTCAGGCTCAGAAG | (Li et al., 2025) |
| *Casp1-R* | TGCGGCTTGACTTGTCCATTATTG |  |
| *p38-F* | GAATGGTGCTCTCTGGTTGGAAGG | (Gao et al., 2020) |
| *p38-R* | TCTTCGGTCACGGAGTCCTTAGC |  |
| *ERK-F* | CCAAGAACCTCGCCATCCTGAAAG |  |
| *ERK-R* | CAGACGACACCACGCCATAAGC |  |
| *JNK-F* | CACAAGATGGCGGCAGGAAGATG |  |
| *JNK-R* | TGTAGGGCGATCAGGAAGGTTCTC |  |
| *Nrf2-F* | GTGTGCGTGTGGACCTGGATTC |  |
| *Nrf2-R* | GAAGGCTGTGGAACTGAGTCTGTG |  |
| *HIF-1-F* | CGCTTGACGGAACTTCGGAGAC |  |
| *HIF-1-R* | CCTTCGACACCTTGCAGCATCC |  |
| *VEGFA-F* | CCTGCGTGCCACTGATGAGATG |  |
| *VEGFA-R* | ATTGTGATGTTGTGCCATGCTGTTG |  |

| **Table S3** Primary and secondary antibodies used for western blotting | | |
| --- | --- | --- |
| **Antibodies name** | **Cat#** | **Company** |
| NLRP12 | DF14960 | Affinity Bioscience, USA |
| IL-β | A11370 | Wuhan Ab-clonal Biotechnology Co., Ltd. |
| TNFα | AF7014 | Affinity Bioscience, USA |
| IL-6 | DF6087 | Affinity Bioscience, USA |
| Caspase-1 | A0964 | Wuhan Ab-clonal Biotechnology Co., Ltd. |
| IRF7 | DF7503 | Affinity Bioscience, USA |
| Bcl-xL | AF6414 | Affinity Bioscience, USA |
| Anti-rabbit antibodies | AS014 | Wuhan Ab-clonal Biotechnology Co., Ltd. |

| **Table S4** Transcriptome sequencing and library statistics results of C57BL/6 mice | | | | | |
| --- | --- | --- | --- | --- | --- |
| **Sample** | **Raw Reads** | **Clean Reads** | **Q20(%)** | **Q30(%)** | **GC Content (%)** |
| Control_Female_1 | 41734840 | 40398602 | 98.24 | 94.56 | 46.92 |
| Control_Female_2 | 49929884 | 48793562 | 98.41 | 95.09 | 46.8 |
| Control_Female_3 | 41372042 | 40477742 | 98.33 | 94.77 | 46.23 |
| Control_Male_1 | 44651152 | 43825346 | 98.25 | 94.6 | 46.12 |
| Control_Male_2 | 44429996 | 43506886 | 98.26 | 94.54 | 47.75 |
| Control_Male_3 | 47293254 | 45857538 | 98.31 | 94.79 | 46.41 |
| PFESA_Female_1 | 47418970 | 46664838 | 98.3 | 94.73 | 46.63 |
| PFESA_Female_2 | 42105816 | 41247714 | 98.38 | 94.97 | 47.28 |
| PFESA_Female_3 | 48801360 | 47915182 | 98.3 | 94.74 | 46.78 |
| PFESA_Male_1 | 54166416 | 52984628 | 98.36 | 94.93 | 47.1 |
| PFESA_Male_2 | 47719114 | 46665014 | 98.54 | 95.45 | 47.03 |
| PFESA_Male_3 | 49758850 | 48749440 | 98.46 | 95.26 | 46.26 |

Fan, S., Popli, S., Chakravarty, S., Chakravarti, R., & Chattopadhyay, S. (2024). Non-transcriptional IRF7 interacts with NF-κB to inhibit viral inflammation. *Journal of Biological Chemistry*, *300*(4).

Gao, H., Cao, Y., Xia, H., Zhu, X., & Jin, Y. (2020). CYP4A11 is involved in the development of nonalcoholic fatty liver disease via ROS-induced lipid peroxidation and inflammation. *International journal of molecular medicine*, *45*(4), 1121-1129.

Ghaemi, S., Arefian, E., Valojerdi, R. R., Soleimani, M., Moradimotlagh, A., & Adegani, F. J. (2020). Inhibiting the expression of anti-apoptotic genes BCL2L1 and MCL1, and apoptosis induction in glioblastoma cells by microRNA-342. *Biomedicine & Pharmacotherapy*, *121*, 109641.

Li, X., Zhou, G., Sun, X., Qu, S., Lai, H., Wu, Y., Li, D., Liu, L., Zhang, G., & Yang, J. (2024). NLRP12 senses the SARS-CoV-2 membrane protein and promotes an inflammatory response. *The Journal of Infectious Diseases*, *229*(3), 660-670.

Li, Y., Zhang, Z., Zhao, P., & Qiao, P. (2025). Brucella-Induced activation of AIM2 inflammasome and Caspase-1 enhances IL-18 secretion in THP-1 cells. *Srpski arhiv za celokupno lekarstvo*, *153*(1-2), 35-41.
